# Anticholinergic drug burden and incident cardiovascular events: a population-based study

**DOI:** 10.1186/s12916-026-04751-w

**Published:** 2026-02-28

**Authors:** Nanbo Zhu, Maria Eriksdotter, Bahira Shahim, Kristina Johnell, Sara Garcia-Ptacek, Juan-Jesus Carrero, Hong Xu

**Affiliations:** 1 Division of Clinical Geriatrics, Department of Neurobiology, Care Sciences and Society, Karolinska Institutet, Stockholm, Sweden; 2 Theme Inflammation and Aging, Karolinska University Hospital, Stockholm, Sweden; 3 Division of Cardiology, Department of Medicine, Karolinska Institutet, Stockholm, Sweden; 4 Department of Medical Epidemiology and Biostatistics, Karolinska Institutet, Stockholm, Sweden; 5 Division of Nephrology, Department of Clinical Sciences, Danderyd Hospital, Stockholm, Sweden

**Keywords:** Anticholinergic drug burden, Cardiovascular disease, Cohort study

## Abstract

**Background:**

Drugs with anticholinergic properties are commonly used in older adults for various medical conditions, but the long-term cardiovascular consequences of cumulative exposure have not been well established. This study aims to examine whether cumulative anticholinergic drug burden is associated with incident cardiovascular events.

**Methods:**

The large population-based cohort study included 508,273 Stockholm residents aged ≥ 45 years on January 1, 2008, who had no history of major cardiovascular diseases, with follow-up until December 31, 2021. Anticholinergic burden was assessed using the Anticholinergic Cognitive Burden scale and quantified as annual consumption in defined daily doses (DDDs). Inverse probability-weighted Cox proportional hazards models were used to estimate the weighted hazard ratio (HR) and 95% confidence interval (CI) for the associations between both baseline and time-varying exposure and incident cardiovascular events, overall and by disease subtypes.

**Results:**

A total of 118,266 incident cardiovascular events were recorded during a median follow-up of 14.0 years. Higher levels of anticholinergic drug exposure were significantly associated with an increased risk of cardiovascular events after adjusting for sociodemographic, lifestyle, and clinical risk factors in both baseline and time-updated models, with stronger associations observed in the latter. In the time-updated model, the HR (95% CI) increased with annual cumulative exposure: 1.16 (1.13, 1.20) for 1–89 DDDs, 1.31 (1.28, 1.34) for 90–364 DDDs, and 1.71 (1.67, 1.76) for ≥ 365 DDDs. A significant dose–response relationship was observed across event subtypes. In the highest exposure group, the HR (95% CI) was 2.70 (2.57, 2.84) for heart failure, 2.17 (2.08, 2.27) for arrhythmias, 1.48 (1.34, 1.63) for artery disease, 1.32 (1.21, 1.43) for venous thromboembolism, 1.46 (1.37, 1.55) for myocardial infarction, and 1.32 (1.25, 1.39) for cerebrovascular disease. Results were consistent in subgroups and sensitivity analyses.

**Conclusions:**

These findings highlight the potential cardiovascular harms of anticholinergic drug burden in middle-aged and older adults and underscore the need for careful prescribing and monitoring of such medications.

**Supplementary Information:**

The online version contains supplementary material available at 10.1186/s12916-026-04751-w.

## Background

Drugs with anticholinergic properties, which inhibit the actions of acetylcholine both centrally and peripherally, are frequently prescribed to older adults for a range of medical conditions [1]. While agents such as antispasmodics are usually used for their anticholinergic properties, many other medications, including antihistamines, antidepressants, and antipsychotics, can unintentionally induce anticholinergic adverse effects beyond their primary mechanism of action [2]. Cumulative use of such medications, termed anticholinergic burden, is typically assessed using expert consensus-based scales [3]. Despite the growing recognition of negative health outcomes, such as cognitive impairment and fractures [4, 5], related to high anticholinergic burden, evidence on its relationship with cardiovascular disease (CVD) remains limited.

Cholinergic signaling, mediated by acetylcholine, plays a crucial physiological role in cardiovascular regulation [6]. In the heart, acetylcholine released from parasympathetic nerves primarily targets M_2_ muscarinic receptors to reduce heart rate and contractility [7], which is essential for maintaining cardiovascular homeostasis by counterbalancing sympathoexcitation [8]. An imbalance between sympathetic and parasympathetic activity, known as autonomic dysfunction, has been implicated in several CVDs [9–12]. Recent studies have also uncovered an endogenous cholinergic system within cardiomyocytes that contributes to important cardiac function [13, 14]. Anticholinergic drugs may disrupt these pathways and impair cardiovascular regulation.

To date, only a few cohort studies have reported associations between anticholinergic burden and increased stroke [15–17] or general cardiovascular risk [18, 19], but mostly assessed exposure only at baseline and did not differentiate types of CVDs. In addition, a recent increase in anticholinergic burden has been linked to acute cardiovascular events, including myocardial infarction, stroke, arrhythmias, conduction disorders, syncope, and cardiovascular death [20]. Conversely, observational studies suggest that enhancing cholinergic signaling with cholinesterase inhibitors may have cardioprotective effects [21–23]. Taken together, cholinergic pathways might be a new target for the prevention and treatment of CVDs [24, 25].

This study aimed to examine the association between anticholinergic burden and incident cardiovascular events in a large general population cohort from Stockholm, Sweden. We hypothesized that greater exposure to anticholinergic drugs would be associated with an elevated incidence of overall CVDs and explored associations with specific types of CVDs.

## Methods

### Data source

This study used data from the Stockholm CREAtinine Measurements (SCREAM) project [26], a health care utilization cohort that included all residents of Stockholm, Sweden, during 2006–2021. The Stockholm region, with a population of 2.3 million residents as of 2021, provides universal healthcare through a single, unified health system. Administrative databases (VAL) [27], containing detailed information on demographics, healthcare utilization, clinical diagnoses, and therapeutic/surgical procedures, were enriched with laboratory tests performed during routine care. Using unique personal identification numbers, these data were linked to the Prescribed Drug Register [28], Cause of Death Register [29], and Longitudinal Integrated Database for Health Insurance and Labor Market Studies [30], which provide information on dispensed medications, vital status, and socioeconomic status, respectively. Data were linked and de-identified by the Swedish National Board of Health and Welfare, with minimal or no loss to follow-up. The study was approved by the regional ethical review board and the Swedish National Board of Health and Welfare (Dnr: 2017/793–31), with informed consent waived for register-based research according to Swedish law (Act [2003:460]). We have followed the Reporting of Studies Conducted Using Observational Routinely Collected Health Data for Pharmacoepidemiology (RECORD-PE) guideline [31].

### Study design

**Fig. 1:**
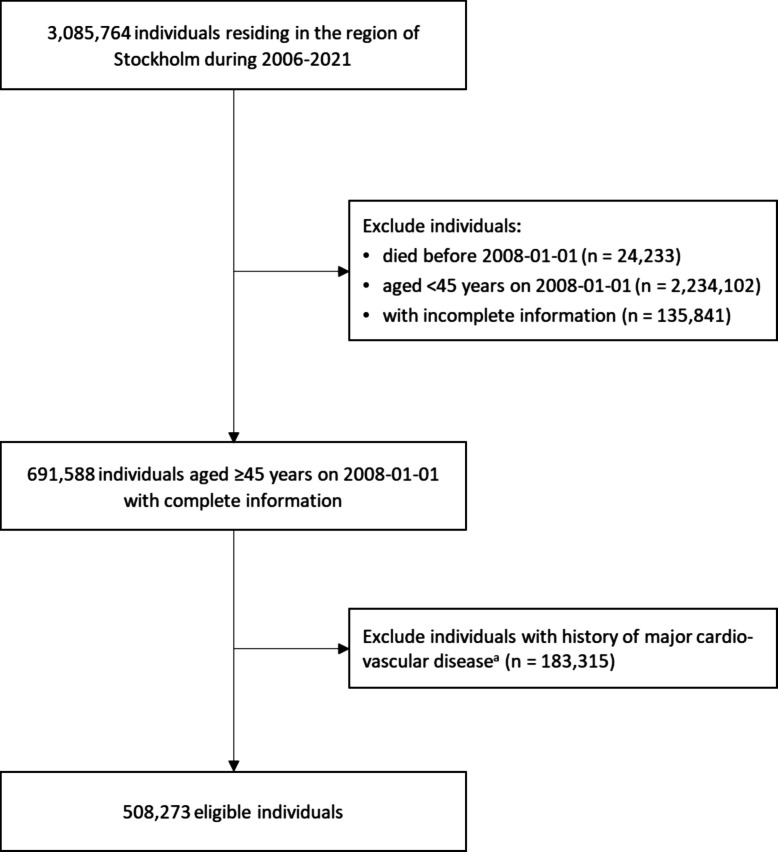
Flowchart of the sample selection. ^a^Individuals who had hypertension but did not have hypertensive heart failure or any other cardiovascular diseases were eligible for inclusion

We conducted a retrospective cohort study of all residents in Stockholm aged ≥ 45 years on January 1, 2008, without a prior history of major CVDs. Individuals with uncomplicated hypertension (i.e., hypertension without hypertensive heart failure or any other CVDs) were eligible for inclusion. A graphical depiction of the study design is shown in Additional file 1: Fig. S1. Given the establishment of the Swedish Prescribed Drug Register in July 2005 [28], we set the baseline (i.e., cohort entry) as January 1, 2008, and required individuals to have been continuously residing in the region since 2006 and for a minimum of 2 years. A flowchart illustrating the sample selection is presented in Fig. 1.

### Exposures

Drugs with anticholinergic properties were identified based on the Anticholinergic Cognitive Burden (ACB) scale [32], the most widely used tool for measuring anticholinergic burden and the highest‐ranked instrument in a quality assessment of available scales [3]. This three-level scale classifies drugs according to their anticholinergic potency (Additional file 1: Table S1). Drugs not on the list are considered to have no anticholinergic effects.

The ACB scale was originally developed to quantify the anticholinergic activity of medications in terms of cognitive adverse effects and does not incorporate cumulative exposure [33]. However, this feature may not be directly applicable when evaluating drugs’ peripheral anticholinergic effect on the cardiovascular system. In this study, the primary measure of anticholinergic use was the annual consumption of drugs with anticholinergic properties, irrespective of their ACB score, which might be more biologically relevant. At baseline, we calculated the cumulative defined daily doses (DDDs) for all drugs listed in the ACB scale that had been dispensed over the previous calendar year. The DDD represents the assumed average maintenance dose per day for a drug used for its main indication in adults [34]. Annual total number of DDDs was categorized into 0, 1–89, 90–364, or ≥ 365 DDDs for clinical interpretability [35]. Since drug exposure varied over time, for time-updated analyses, we assessed the annual DDDs at study entry and updated the exposure using 1-year sliding windows, anchored at the beginning of each calendar year throughout follow-up.

We used the total score of ACB drugs as a secondary exposure to enhance comparability with prior studies. The baseline value was calculated by summing the points of all distinct ACB drugs dispensed during the year prior to baseline. This exposure was categorized into 0, 1, 2–3, or ≥ 4, in line with previous research [16, 18], and was similarly reassessed annually in the time-updated analyses.

### Outcomes

The primary outcome was any incident cardiovascular event resulting in hospitalization or death, identified using the main and first contributory hospital discharge diagnoses, as well as the underlying cause of death. Specific types of cardiovascular events, including myocardial infarction, arrhythmias, heart failure, cerebrovascular disease, arterial disease, and venous thromboembolism (Additional file 1: Table S2), were analyzed as secondary outcomes. Follow-up started on the index date and ended at the earliest occurrence of a cardiovascular event, death, emigration from the region, or study end (December 31, 2021).

### Covariates

Potential confounders were cardiovascular risk factors and indications for anticholinergic drugs, including sociodemographic factors, healthcare utilization in the previous year, surrogates for lifestyle factors, lifetime diagnoses of physical, neurological, and psychiatric conditions, and use of non-anticholinergic medications within the previous year (see covariate list in Table 1). Definitions of medical conditions and medications are provided in Additional file 1: Table S3. Covariates were updated yearly in the time-varying exposure analyses, with age and sex retained as fixed characteristics. The VAL databases contain information on primary care consultations since 2003 and outpatient/inpatient care visits since 1997 [27], providing sufficient time to ascertain comorbidities.

**Table 1:** Characteristics according to annual DDDs of anticholinergic drugs at baseline

Characteristics	Overall *N* = 508,273	0 *N* = 365,398	1–89 *N* = 52,000	90–364 *N* = 63,729	≥ 365 *N* = 27,146
**Age, years, median (IQR)**	58.9 (51.6–66.6)	58.0 (50.9–65.4)	59.3 (51.8–67.5)	62.3 (54.8–71.1)	62.7 (55.0–72.1)
**Female**	270,441 (53.2)	182,777 (50.0)	31,774 (61.1)	39,147 (61.4)	16,743 (61.7)
**Disposable income, × 100 SEK, median (IQR)**^**a**^	2080 (1369–2934)	2192 (1452–3049)	1942 (1323–2759)	1801 (1265–2625)	1520 (1183–2264)
**Educational attainment**^**a**^					
Compulsory education	104,848 (21.0)	69,755 (19.4)	11,389 (22.2)	16,084 (25.7)	7620 (28.8)
Upper secondary education	206,333 (41.3)	147,140 (41.0)	21,338 (41.7)	26,425 (42.2)	11,430 (43.2)
University/college	187,964 (37.7)	141,921 (39.6)	18,469 (36.1)	20,168 (32.2)	7406 (28.0)
**Marital status**^**a**^					
Single	199,270 (39.4)	143,122 (39.4)	20,888 (40.2)	23,404 (36.8)	11,856 (43.7)
Married	268,191 (53.0)	197,068 (54.2)	26,401 (50.8)	32,828 (51.5)	11,894 (43.8)
Widowed	38,717 (7.6)	23,213 (6.4)	4675 (9.0)	7450 (11.7)	3379 (12.5)
**Any outpatient specialist visits in the past year**	176,969 (34.8)	103,646 (28.4)	27,867 (53.6)	29,937 (47.0)	15,519 (57.2)
**Any hospitalization in the past year**	35,643 (7.0)	16,752 (4.6)	7444 (14.3)	7244 (11.4)	4203 (15.5)
**Tobacco-related disorders**	4961 (1.0)	2656 (0.7)	891 (1.7)	886 (1.4)	528 (1.9)
**Alcohol-related disorders**	12,564 (2.5)	6352 (1.7)	1850 (3.6)	2402 (3.8)	1960 (7.2)
**Obesity diagnosis**	12,933 (2.5)	6229 (1.7)	1819 (3.5)	3046 (4.8)	1839 (6.8)
**Physical conditions**					
Hypertension	138,366 (27.2)	59,896 (16.4)	18,257 (35.1)	42,027 (65.9)	18,186 (67.0)
Chronic pain	84,731 (16.7)	52,211 (14.3)	12,861 (24.7)	13,503 (21.2)	6156 (22.7)
Back pain	66,932 (13.2)	40,274 (11.0)	10,808 (20.8)	10,748 (16.9)	5102 (18.8)
Osteoarthritis	49,327 (9.7)	29,481 (8.1)	6949 (13.4)	8856 (13.9)	4041 (14.9)
Cancer	34,681 (6.8)	20,902 (5.7)	4891 (9.4)	6126 (9.6)	2762 (10.2)
Diabetes mellitus	33,878 (6.7)	17,732 (4.9)	3842 (7.4)	7552 (11.9)	4752 (17.5)
Lung disease	30,655 (6.0)	16,651 (4.6)	4287 (8.2)	6364 (10.0)	3353 (12.4)
Dyslipidemia	29,516 (5.8)	16,060 (4.4)	3475 (6.7)	6991 (11.0)	2990 (11.0)
Kidney disease	24,972 (4.9)	10,954 (3.0)	3235 (6.2)	6494 (10.2)	4289 (15.8)
Rheumatic disease	17,907 (3.5)	10,629 (2.9)	2421 (4.7)	3268 (5.1)	1589 (5.9)
Gastroesophageal reflux disease	14,326 (2.8)	8009 (2.2)	2339 (4.5)	2627 (4.1)	1351 (5.0)
Urinary incontinence	11,983 (2.4)	5928 (1.6)	1797 (3.5)	2653 (4.2)	1605 (5.9)
Vestibular disorder	11,329 (2.2)	7072 (1.9)	1575 (3.0)	1922 (3.0)	760 (2.8)
Irritable bowel syndrome	8559 (1.7)	4621 (1.3)	1591 (3.1)	1570 (2.5)	777 (2.9)
Liver disease	7431 (1.5)	4272 (1.2)	1037 (2.0)	1294 (2.0)	828 (3.1)
Peptic ulcer disease	5089 (1.0)	2821 (0.8)	764 (1.5)	933 (1.5)	571 (2.1)
Inflammatory bowel disease	4810 (0.9)	2940 (0.8)	683 (1.3)	807 (1.3)	380 (1.4)
**Neurological/psychiatric conditions**					
Depression	29,792 (5.9)	13,871 (3.8)	5473 (10.5)	6105 (9.6)	4343 (16.0)
Migraine/headache	29,395 (5.8)	16,946 (4.6)	4949 (9.5)	5138 (8.1)	2362 (8.7)
Sleep disorders	22,578 (4.4)	12,005 (3.3)	4035 (7.8)	4341 (6.8)	2197 (8.1)
Stress-related disorders	20,637 (4.1)	11,891 (3.3)	3645 (7.0)	3489 (5.5)	1612 (5.9)
Substance use disorder	17,780 (3.5)	8950 (2.4)	2775 (5.3)	3300 (5.2)	2755 (10.1)
Anxiety disorders	16,668 (3.3)	6527 (1.8)	3359 (6.5)	3728 (5.8)	3054 (11.3)
Psychotic disorders	6973 (1.4)	1840 (0.5)	946 (1.8)	1,828 (2.9)	2359 (8.7)
Transient ischemic attack	4213 (0.8)	2254 (0.6)	570 (1.1)	943 (1.5)	446 (1.6)
Epilepsy	3908 (0.8)	1510 (0.4)	537 (1.0)	1127 (1.8)	734 (2.7)
Parkinson’s disease	1431 (0.3)	726 (0.2)	229 (0.4)	298 (0.5)	178 (0.7)
**Medications**^**b**^					
Number of distinct drugs, median (IQR)	2 (1–5)	2 (0–4)	5 (2–8)	5 (3–9)	7 (4–11)
Nonsteroidal anti-inflammatory drugs	100,992 (19.9)	58,430 (16.0)	17,229 (33.1)	17,298 (27.1)	8035 (29.6)
Anxiolytics, hypnotics, and sedatives	72,655 (14.3)	31,194 (8.5)	13,648 (26.2)	16,882 (26.5)	10,931 (40.3)
Renin-angiotensin system inhibitors	68,793 (13.5)	34,925 (9.6)	8318 (16.0)	17,200 (27.0)	8350 (30.8)
Statins	54,349 (10.7)	27,414 (7.5)	6330 (12.2)	13,747 (21.6)	6858 (25.3)
Proton-pump inhibitors	45,767 (9.0)	21,105 (5.8)	8369 (16.1)	10,298 (16.2)	5995 (22.1)
Antidepressants	37,880 (7.5)	16,435 (4.5)	7712 (14.8)	8467 (13.3)	5266 (19.4)
Diuretics	37,165 (7.3)	16,626 (4.6)	4579 (8.8)	11,282 (17.7)	4678 (17.2)
Calcium channel blockers	35,143 (6.9)	13,799 (3.8)	3772 (7.3)	11,790 (18.5)	5782 (21.3)
Opioids	30,946 (6.1)	14,007 (3.8)	6115 (11.8)	6698 (10.5)	4126 (15.2)
Antiplatelet drugs	30,053 (5.9)	12,889 (3.5)	3873 (7.4)	8812 (13.8)	4479 (16.5)
Beta-blockers	11,728 (2.3)	6361 (1.7)	1834 (3.5)	2115 (3.3)	1418 (5.2)
Antiepileptics	7692 (1.5)	2295 (0.6)	1447 (2.8)	1932 (3.0)	2018 (7.4)
Anti-addiction drugs	7413 (1.5)	3528 (1.0)	1304 (2.5)	1442 (2.3)	1139 (4.2)
Anti-Parkinson drugs	4782 (0.9)	1865 (0.5)	751 (1.4)	1127 (1.8)	1039 (3.8)
Antipsychotics	4650 (0.9)	1351 (0.4)	797 (1.5)	1165 (1.8)	1337 (4.9)

Frequency with percentage was reported unless otherwise specified

*DDD* defined daily dose, *IQR* interquartile range

^a^Disposable income and marital status were missing for 2095 (0.4%) individuals; educational attainment was missing for 9128 (1.8%) individuals

^b^Drugs listed in the Anticholinergic Cognitive Burden scale were excluded from the covariate definition

### Statistical analysis

Characteristics of the study population were summarized as median with interquartile range (IQR) or frequency with percentage, stratified by annual DDDs of anticholinergic drugs at baseline. In the baseline (i.e., time-fixed) exposure analysis, we estimated inverse probability weights (IPW) for the exposure categories (during 2007–2008) using a multinomial logistic regression [36] that included baseline covariates plus pre-baseline exposure (during 2006–2007). Adjustment for past exposure was a statistical approach to mitigate prevalent user bias [37]. The weights were stabilized and trimmed at the 0.5th and 99.5th percentiles to improve precision and reduce the impact of extreme values [38]. Covariate balance was evaluated using standardized mean differences, with values ≤ 0.1 considered adequate balance [38]. We then fitted an IPW-weighted cause-specific Cox model to assess the association between baseline anticholinergic drug exposure and incident cardiovascular events. For comparison, we applied traditional multivariable Cox models, with age as the underlying time scale, to examine this association. In addition, we fitted an IPW-weighted Fine-Gray model as an alternative approach to account for competing risk from non-CVD death.

Alluvial diagrams were used to illustrate changes in drug exposure categories over time, with subsequent analyses focusing on annual DDDs of anticholinergic drugs as a time-varying categorical exposure. To properly account for time-dependent confounding, we estimated IPW at each year using multinomial logistic regression models, conditional on exposure history and time-fixed and time-varying covariates [38]. The weights were multiplied across periods, which helps create a pseudopopulation in which covariate balance between groups is achieved over time and current exposure is no longer affected by past exposure or measured confounders. Weights were stabilized and trimmed at the 0.5th and 99.5th percentiles. We used the marginal structural Cox regression model to examine the association between time-varying anticholinergic drug exposure and incident cardiovascular events.

To assess potential variations in associations by CVD subtypes, we conducted IPW-weighted analyses for specific types of CVDs. The abovementioned analyses were repeated using the total ACB score as the secondary exposure. Hazard ratios (HRs) and 95% confidence intervals (CIs) were reported for all models. As hazards are often non-proportional in medical research, the HRs estimated from Cox models should be interpreted as a weighted average of time-specific HRs over the follow-up [39].

### Sensitivity and subgroup analysis

To test the robustness of our results, several sensitivity analyses focusing on the time-varying anticholinergic drug exposure (annual DDDs) and any incident cardiovascular events were performed. First, to account for the overall increase in medication use among the higher exposure groups, we calculated the number of distinct non-anticholinergic drugs within each exposure assessment window and included it as a time-varying confounder, instead of relying on pre-selected drug categories. Second, to minimize the risk of indication bias, (i) we performed separate analyses in individuals with and without hypertension at baseline; (ii) we excluded cardiovascular-related anticholinergic drugs listed in the ACB scale from the exposure definition. Third, we applied a 1-year washout period and excluded individuals with dispensations of ACB drugs during 2006–2007 to approximate a new-user design, as an alternative approach to address prevalent user bias. Finally, we included CVD diagnoses from all sources (primary, outpatient, and inpatient care) in our outcome definition to improve the sensitivity of event identification.

To explore potential effect modification, we conducted pre-specified subgroup analyses according to sex (female/male) and age (< 55, 55–64, or ≥ 65 years) at baseline, and tested for multiplicative interactions. A two-tailed *P* < 0.05 was chosen as the threshold for statistical significance. All analyses were performed using R version 4.3.2 (R Foundation for Statistical Computing).

## Results

### Population characteristics

The study population consisted of 508,273 individuals aged ≥ 45 years with no prior history of major CVDs (Fig. 1). The median (IQR) age was 58.9 (51.6–66.6) years, and 270,441 (53.2%) individuals were women (Table 1). Among the cohort, 365,398 (71.9%) individuals had no exposure to anticholinergic drugs at baseline, while 10.2%, 12.5%, and 5.3% were classified as having an annual consumption of 1–89, 90–364, and ≥ 365 DDDs of anticholinergic drugs, respectively. Those with higher levels of anticholinergic drug use were generally older, were more likely to be female, and exhibited lower socioeconomic status. Additionally, groups with greater exposure demonstrated a higher prevalence of medical conditions and increased use of non-anticholinergic medications.

Additional file 1: Fig. S2 depicts the changes in exposure categories over the first four years of follow-up. Although the percentage composition across exposure levels remained relatively stable year by year, transitions between groups over time were not uncommon. As shown in the Additional file 1: Fig. S3, drugs classified under the Anatomical Therapeutic Chemical (ATC) group C (cardiovascular system) accounted for the largest proportion of population total DDDs of anticholinergic drugs, followed by group N (nervous system) and group R (respiratory system).

### Anticholinergic burden and incident CVD

During a median follow-up of 14.0 years (IQR: 8.7–14.0), a total of 118,266 incident cardiovascular events were recorded. For the baseline exposure analysis, Additional file 1: Fig. S4 shows the distribution of IPW weights. Covariates for sociodemographic, lifestyle, clinical risk factors, and past anticholinergic exposure were balanced after weighting (Additional file 1: Fig. S5). The weighted HRs (95% CIs) for baseline anticholinergic burden increased from 1.05 (1.02, 1.08) for 1–89 DDDs and 1.05 (1.01, 1.09) for 90–364 DDDs to 1.17 (1.08, 1.27) for ≥ 365 DDDs (Table 2). Similar results were obtained using traditional multivariable Cox models (Additional file 1: Table S4), while estimates were slightly attenuated in the Fine-Gray model (Additional file 1: Table S5).

**Table 2:** Association between anticholinergic burden (annual DDDs) and incident cardiovascular events

	No. of events	Person-year	Incidence rate per 1000 person-years	Weighted HR (95% CI)^a^
**Baseline exposure (DDDs)**				
0	76,004	4,211,926	18.0	1.00
1–89	12,686	565,755	22.4	1.05 (1.02, 1.08)
90–364	19,576	660,411	29.6	1.05 (1.01, 1.09)
≥ 365	10,000	256,643	39.0	1.17 (1.08, 1.27)
**Time-varying exposure (DDDs)**				
0	62,942	3,927,587	16.0	1.00
1–89	15,166	622,336	24.4	1.16 (1.13, 1.20)
90–364	25,435	810,992	31.4	1.31 (1.28, 1.34)
≥ 365	14,723	333,820	44.1	1.71 (1.67, 1.76)

*CI* confidence interval, *DDD* defined daily dose, *HR* hazard ratio

^a^Model was weighted for age, sex, socioeconomic status, healthcare utilization, medical conditions, use of medications other than anticholinergics, and pre-baseline exposure to anticholinergic drugs

**Fig. 2:**
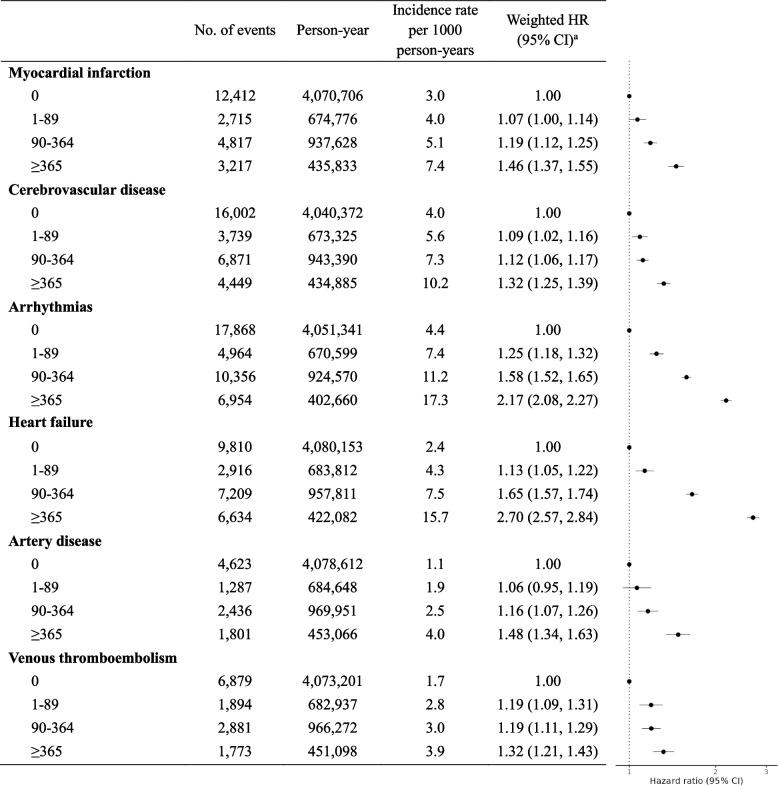
Associations between time-varying anticholinergic burden (annual DDDs) and specific cardiovascular events. Abbreviations: CI, confidence interval; DDD, defined daily dose; HR, hazard ratio. ^a^Models were weighted for age, sex, socioeconomic status, healthcare utilization, medical conditions, use of medications other than anticholinergics, and pre-baseline exposure to anticholinergic drugs

In the time-varying exposure analysis, Additional file 1: Fig. S6 shows the weight distributions over time. Of note, stronger and graded associations were observed compared to the baseline model, with corresponding HRs (95% CIs) of 1.16 (1.13, 1.20), 1.31 (1.28, 1.34), and 1.71 (1.67, 1.76), respectively (Table 2). Figure 2 presents a forest plot illustrating the associations between time-varying anticholinergic burden and specific types of CVDs. A significant dose–response relationship was observed across all CVD subtypes, with notably stronger associations for heart failure and arrhythmias. For example, the weighted HR (95% CI) for individuals exposed to ≥ 365 DDDs of anticholinergic drugs was 2.70 (2.57, 2.84) for heart failure and 2.17 (2.08, 2.27) for arrhythmias, compared to 1.46 (1.37, 1.55) for myocardial infarction and 1.32 (1.25, 1.39) for cerebrovascular disease.

Additional file 1: Tables S6–S7 and Fig. S7 present the results using the total ACB score as a secondary exposure, indicating consistent findings that a greater anticholinergic burden was related to an increased incidence rate of cardiovascular events.

### Sensitivity and subgroup analysis

Table 3 summarizes the results from several sensitivity analyses, all of which were statistically significant and demonstrated a dose–response pattern. First, the association was similar when accounting for the number of non-anticholinergic drugs rather than pre-selected medications. Second, restricting the analysis to individuals with/without hypertension at baseline slightly attenuated the association, whereas excluding cardiovascular-related anticholinergic drugs from the exposure definition had a larger impact. Third, the association remained consistent after excluding individuals with past anticholinergic exposure using a one-year washout period. Finally, incorporating CVD diagnosis from all levels of health care led to a higher incidence rate but weakened the strength of the association.

**Table 3:** Sensitivity analyses of the association between time-varying anticholinergic burden (annual DDDs) and incident cardiovascular events

	No. of events	Person-year	Incidence rate per 1000 person-years	Weighted HR (95% CI)^a^
**Adjust for the number of non-ACB drugs (*N* = 508,273)**				
0	62,942	3,927,587	16.0	1.00
1–89	15,166	622,336	24.4	1.16 (1.12, 1.19)
90–364	25,435	810,992	31.4	1.30 (1.27, 1.33)
≥ 365	14,723	333,820	44.1	1.74 (1.70, 1.79)
**Restrict to individuals without hypertension at baseline (*N* = 369,907)**				
0	46,260	3,308,657	14.0	1.00
1–89	9227	455,993	20.2	1.13 (1.08, 1.17)
90–364	9918	394,460	25.1	1.22 (1.17, 1.27)
≥ 365	4628	150,062	30.8	1.53 (1.45, 1.62)
**Restrict to individuals with hypertension at baseline (*N* = 138,366)**				
0	16,682	618,931	27.0	1.00
1–89	5939	166,343	35.7	1.15 (1.11, 1.20)
90–364	15,517	416,532	37.3	1.25 (1.22, 1.28)
≥ 365	10,095	183,758	54.9	1.63 (1.58, 1.67)
**Exclude cardiovascular-related anticholinergic drugs from the exposure definition (*N* = 508,273)**				
0	83,933	4,434,049	18.9	1.00
1–89	15,136	598,757	25.3	1.08 (1.05, 1.11)
90–364	13,502	465,630	29.0	1.24 (1.20, 1.27)
≥ 365	5695	196,299	29.0	1.34 (1.29, 1.39)
**Exclude individuals with past ACB use (*N* = 367,275)**				
0	52,982	3,421,381	15.5	1.00
1–89	9992	419,560	23.8	1.14 (1.09, 1.18)
90–364	10,464	310,020	33.8	1.26 (1.21, 1.31)
≥ 365	3509	69,876	50.2	1.66 (1.54, 1.78)
**Include CVD diagnosis from all healthcare sources (*N* = 508,273)**				
0	130,552	3,520,252	37.1	1.00
1–89	27,721	515,465	53.8	1.15 (1.12, 1.17)
90–364	39,179	646,194	60.6	1.24 (1.22, 1.26)
≥ 365	18,825	254,286	74.0	1.48 (1.45, 1.51)

*ACB* anticholinergic burden, *CI* confidence interval, *CVD* cardiovascular disease, *DDD* defined daily dose, *HR* hazard ratio

^a^Models were weighted for age, sex, socioeconomic status, healthcare utilization, medical conditions, use of medications other than anticholinergics, and pre-baseline exposure to anticholinergic drugs

The association between anticholinergic drug exposure and incident cardiovascular events was consistently observed in both women and men (Additional file 1: Table S8), as well as in younger individuals and older adults (Additional file 1: Table S9).

## Discussion

In this large population-based cohort study among middle-aged and older adults, we found an increased risk of incident cardiovascular events associated with cumulative exposure to anticholinergic drugs, showing a dose–response pattern. Significant associations were observed for each specific type of CVD, with a more pronounced association for heart failure and arrhythmias than for myocardial infarction, cerebrovascular disease, arterial disease, and venous thromboembolism.

### Comparison with previous studies

Although it is well recognized that anticholinergic drugs suppress parasympathetic control of the heart, possibly resulting in tachycardia, few studies have examined the cardiovascular safety associated with anticholinergic burden [15–19]. Myint et al. reported a significant dose–response relationship between baseline anticholinergic burden, assessed by self-reported medication use, and incidence of overall CVDs in the EPIC-Norfolk cohort [18]. Similarly, a Danish study of geriatric outpatients indicated an association between baseline anticholinergic burden and risk of major adverse cardiovascular events [19]. However, assessing only baseline exposure is a key limitation in both studies, as our research demonstrated that anticholinergic burden fluctuates over time, and failure to account for time-varying exposure underestimated the association. Unlike our investigation of cardiovascular risk in relation to cumulative anticholinergic burden, Huang et al. employed a case-case-time-control design to capture the transient effect while mitigating protopathic bias [20]. Their study established a link between acute cardiovascular events and elevated anticholinergic burden in the 30 days preceding the index event, indicating that older adults with an ACB score of ≥ 3 had a twofold increased risk compared to those with a score of 0. These findings raise concerns about the cardiovascular safety of even short-term exposure to a high anticholinergic burden.

Three cohort studies have focused exclusively on stroke, all of which reported significant findings [15–17]. However, no prior studies have evaluated the long-term risks across subtypes of CVDs. Our findings showed a stronger association with heart failure and arrhythmias, conditions more closely related to autonomic dysfunction [11, 12]. The aforementioned study observed a similar acute effect on myocardial infarction, stroke, and arrhythmias [20], whereas our study design was better suited for examining chronic rather than acute events, which may partially explain the discrepancy.

Our hypothesis and findings are supported by biological plausibility. First, anticholinergic drugs inhibit the parasympathetic nervous system, leading to increased heart rate and blood pressure. This disruption in autonomic regulation is reflected by reduced heart rate variability [40, 41], a well-established marker linked to adverse cardiovascular outcomes [42]. Second, certain anticholinergic drugs, such as antipsychotics and long-acting muscarinic antagonists, exhibit pro-ischemic and pro-arrhythmic properties [43, 44], which may contribute to the occurrence of ischemic events and arrhythmias. Third, the cholinergic anti-inflammatory pathway has been implicated [45], and a high anticholinergic burden is associated with elevated levels of inflammation [46], which has a critical role in the pathophysiology of CVDs. Fourth, the discovery of cardiac non-neuronal cholinergic system offers novel insights into the prevention and treatment of CVDs. This system controls electrical conduction and heart rhythm [14], and is thought to sustain or amplify neuronal cholinergic effects, suppress cardiac hypertrophy, and regulate cardiac energy metabolism [13, 25]. Importantly, defects in the cardiomyocyte cholinergic system have been associated with fatal ventricular arrhythmias in both patients and mice [14]. Furthermore, animal experiments suggest that activation of the system protects against hypoxia/reoxygenation injury and cardiovascular complications [47, 48].

Nevertheless, several potential sources of bias warrant discussion. Anticholinergic drugs classified within ATC groups corresponding to the cardiovascular, respiratory, and nervous systems accounted for most of the population total DDDs. Excluding cardiovascular-related anticholinergic drugs from the exposure definition attenuated the association, though it remained statistically significant. Despite the restriction to individuals without a history of major CVDs and extensive covariate adjustment, residual confounding by indication and disease severity, including hypertension, respiratory conditions, and psychiatric disorders, cannot be fully ruled out, particularly given the complexity of the overall anticholinergic burden. In addition, some anticholinergic drugs have other pharmacological actions, such as QT prolongation among certain psychotropic medications [44], which could also be related to CVD. Further investigation of drug class-specific associations may allow for more refined control of bias and help to clarify the observed association.

### Implications of the study

Anticholinergic burden was initially recognized for its detrimental impact on cognition in older adults [4], whereas our study, along with a few others [15–20], highlights its adverse effects on the cardiovascular system. Considering the substantial global burden of CVDs, our findings emphasize the need for healthcare providers to be aware of patients’ overall anticholinergic burden and its potential cardiovascular side effects. While this study does not imply that anticholinergic drugs should always be avoided, minimizing their use, through alternative treatments or dose adjustments, may help mitigate cardiovascular risk. Furthermore, deprescribing anticholinergics has been proposed as a strategy to enhance drug safety by reducing unnecessary drug use [49]. One observational study found that a reduced anticholinergic burden 6 months after baseline was not associated with a lower risk of major adverse cardiovascular events [19], but it lacked a precise definition of deprescribing. Future intervention studies evaluating the effect of deprescribing anticholinergics on cardiovascular events are warranted.

Given the growing evidence of the cardioprotective effects of acetylcholine from both neuronal and non-neuronal sources [25, 50], targeting the cholinergic system has emerged as a promising therapeutic approach for CVDs. Interestingly, cholinesterase inhibitors, which have the opposite effect to anticholinergic drugs, have been found to attenuate cardiac and coronary artery remodeling in rats with chronic heart failure [51], and are associated with a lower risk of cardiovascular events in population-based cohort studies involving patients with Alzheimer’s disease [21–23]. Further studies are needed to evaluate the effectiveness and safety of cholinesterase inhibitors in populations without Alzheimer’s disease.

### Strengths and limitations

Strengths of this study include a large, well-characterized sample from the general population, covering the complete universal tax-funded healthcare of the Stockholm region. Additionally, the use of validated measures to assess anticholinergic burden and determine subtypes of CVDs enabled a thorough investigation. Several limitations should be acknowledged. First, given the observational nature of this study and the complex components of anticholinergic burden, we were unable to establish a causal relationship between anticholinergic burden and cardiovascular risk. The observed dose–response pattern may partly result from residual or unmeasured confounding despite extensive adjustment for covariates. Future experimental research elucidating the effect of clinically used anticholinergic drugs on the cardiac cholinergic system, along with interventional studies evaluating the effect of deprescribing anticholinergics, will provide evidence for causality. Second, exposure misclassification is inevitable, as no consensus exists on the optimal method for assessing anticholinergic burden and current scales were originally developed for cognitive outcomes instead of peripheral anticholinergic effects. Moreover, individuals may not strictly adhere to their prescribed medication regimen, and our register data did not capture anticholinergics sold over the counter or those administered in hospitals. Third, while some ascertainment bias of comorbidities is possible, the long pre-baseline period used to capture these conditions likely mitigates this concern. Similarly, ascertainment bias of CVD and its subtypes cannot be ruled out; however, sensitivity analyses using diagnoses from all health care levels also showed a dose–response relationship. Finally, this study focused on incident events and excluded individuals with preexisting CVD, who represent a distinct clinical group requiring further investigation. Generalizing our findings to other settings should be approached with caution, as prescribing and diagnostic practices vary across healthcare systems.

## Conclusions

This study found an increased risk of incident cardiovascular events associated with higher anticholinergic burden in middle-aged and older adults, exhibiting a dose–response pattern. Significant associations were observed for each specific type of CVD, especially pronounced for heart failure and arrhythmias. Given the widespread use of anticholinergic drugs, healthcare providers should be more vigilant about these potential effects, closely monitor for cardiovascular risks, and consider reducing unnecessary drug use.

## Supplementary Information

Additional file 1: Tables S1–S9. Table S1. List of drugs included in the Anticholinergic Cognitive Burden scale. Table S2. List of ICD-10 codes used to identify cardiovascular events. Table S3. List of ICD-10 codes and ATC codes used to define covariates. Table S4. Association between baseline anticholinergic burden and incident cardiovascular events, using conventional multivariable Cox models. Table S5. Competing risk analysis of baseline anticholinergic burden and incident cardiovascular events. Table S6. Characteristics according to the total ACB score at baseline. Table S7. Association between anticholinergic burden and incident cardiovascular events. Table S8. Association between time-varying anticholinergic burden and incident cardiovascular events, stratified by sex. Table S9. Association between time-varying anticholinergic burden and incident cardiovascular events, stratified by age groups at baseline. Figures S1–S7. Figure S1. Graphical depiction of the study design. Figure S2. Alluvial plots illustrating the changes in drug exposure categories over time. Figure S3. Distribution of population total DDDs of anticholinergic drugs by ATC groups. Figure S4. Distribution of baseline inverse probability weights. Figure S5. Covariate balance before and after baseline inverse probability weighting. Figure S6. Distribution of time-varying inverse probability weights. Figure S7. Associations between time-varying anticholinergic burden and specific cardiovascular events.

## Data Availability

The data may be shared on reasonable request to Prof. Carrero (juan.jesus.carrero@ki.se) for academic research collaborations that comply with the General Data Protection Regulation as well as national and institutional ethics regulations and standards.

## Abbreviations

ACB: Anticholinergic Cognitive Burden
ATC: Anatomical Therapeutic Chemical
CI: Confidence interval
CVD: Cardiovascular disease
DDD: Defined daily dose
HR: Hazard ratio
IPW: Inverse probability weight
IQR: Interquartile range
