# Supplementary material for: Anticholinergic drug burden and incident cardiovascular events: a population-based study
Source: BMC Med. 2026 Feb 28;24:200. doi: 10.1186/s12916-026-04751-w (PMC13059147; doi:10.1186/s12916-026-04751-w)
Supplement: Supplementary file 1 — Additional file 1: Tables S1–S9. Table S1. List of drugs included in the Anticholinergic Cognitive Burden scale. Table S2. List of ICD-10 codes used to identify cardiovascular events. Table S3. List of ICD-10 codes and ATC codes used to define covariates. Table S4. Association between baseline anticholinergic burden and incident cardiovascular events, using conventional multivariable Cox models. Table S5. Competing risk analysis of baseline anticholinergic burden and incident cardiovascular events. Table S6. Characteristics according to the total ACB score at baseline. Table S7. Association between anticholinergic burden and incident cardiovascular events. Table S8. Association between time-varying anticholinergic burden and incident cardiovascular events, stratified by sex. Table S9. Association between time-varying anticholinergic burden and incident cardiovascular events, stratified by age groups at baseline. Figures S1–S7. Figure S1. Graphical depiction of the study design. Figure S2. Alluvial plots illustrating the changes in drug exposure categories over time. Figure S3. Distribution of population total DDDs of anticholinergic drugs by ATC groups. Figure S4. Distribution of baseline inverse probability weights. Figure S5. Covariate balance before and after baseline inverse probability weighting. Figure S6. Distribution of time-varying inverse probability weights. Figure S7. Associations between time-varying anticholinergic burden and specific cardiovascular events. [file 12916_2026_4751_MOESM1_ESM.docx]

**Anticholinergic Drug Burden and Incident Cardiovascular Events: A Population-based Study**

**Supplementary Materials**

[Table S1. List of drugs included in the Anticholinergic Cognitive Burden scale 2](#_Toc221266441)

[Table S2. List of ICD-10 codes used to identify cardiovascular events 5](#_Toc221266442)

[Table S3. List of ICD-10 codes and ATC codes used to define covariates 6](#_Toc221266443)

[Table S4. Association between baseline anticholinergic burden (annual DDDs) and incident cardiovascular events, using conventional multivariable Cox models 8](#_Toc221266444)

[Table S5. Competing risk analysis of baseline anticholinergic burden (annual DDDs) and incident cardiovascular events 9](#_Toc221266445)

[Table S6. Characteristics according to the total ACB score at baseline 10](#_Toc221266446)

[Table S7. Association between anticholinergic burden (ACB score) and incident cardiovascular events 13](#_Toc221266447)

[Table S8. Association between time-varying anticholinergic burden (annual DDDs) and incident cardiovascular events, stratified by sex 14](#_Toc221266448)

[Table S9. Association between time-varying anticholinergic burden (annual DDDs) and incident cardiovascular events, stratified by age groups at baseline 15](#_Toc221266449)

[Figure S1. Graphical depiction of the study design 16](#_Toc221266450)

[Figure S2. Alluvial plots illustrating the changes in drug exposure categories over time 17](#_Toc221266451)

[Figure S3. Distribution of population total DDDs of anticholinergic drugs by ATC groups 18](#_Toc221266452)

[Figure S4. Distribution of baseline inverse probability weights 19](#_Toc221266453)

[Figure S5. Covariate balance before and after baseline inverse probability weighting 20](#_Toc221266454)

[Figure S6. Distribution of time-varying inverse probability weights 21](#_Toc221266455)

[Figure S7. Associations between time-varying anticholinergic burden (ACB score) and specific cardiovascular events 22](#_Toc221266456)

## Table S1. List of drugs included in the Anticholinergic Cognitive Burden scale

| **Generic name** | **Score** | **ATC codes** |
| --- | --- | --- |
| Alimemazine | 1 | R06AD01 |
| Alprazolam | 1 | N05BA12 |
| Alverine | 1 | A03AX08, A03AX58 |
| Aripiprazole | 1 | N05AX12 |
| Asenapine | 1 | N05AH05 |
| Atenolol | 1 | C07AB03, C07AB11, C07BB03, C07CB03, C07CB53, C07DB01, C07FB03 |
| Bupropion | 1 | N06AX12, A08AA62 |
| Captopril | 1 | C09AA01, C09BA01 |
| Cetirizine | 1 | R06AE07 |
| Chlortalidone | 1 | C03BA04, C03BB04, C03EA06 |
| Cimetidine | 1 | A02BA01, A02BA51 |
| Clidinium | 1 | A03CA02 |
| Clorazepate | 1 | N05BA05 |
| Codeine | 1 | R05DA04, R05DA12, N02AA08, N02AA58, N02AA59, N02AA79, N02AJ01, N02AJ02, N02AJ03, N02AJ06, N02AJ07, N02AJ08, N02AJ09 |
| Colchicine | 1 | M04AC01, L01CC |
| Desloratadine | 1 | R06AX27 |
| Diazepam | 1 | N05BA01, N05BA17 |
| Digoxin | 1 | C01AA05, C01AA02, C01AA08, C01AA52 |
| Dipyridamole | 1 | B01AC07 |
| Disopyramide | 1 | C01BA03 |
| Fentanyl | 1 | N01AH01, N01AH51, N02AB03 |
| Fluvoxamine | 1 | N06AB08 |
| Furosemide | 1 | C03CA01, C03CB01, C03EB01 |
| Haloperidol | 1 | N05AD01 |
| Hydralazine | 1 | C02DB02, C02DB01, C02LG01, C02LG02, C02LG51 |
| Hydrocortisone | 1 | H02AB09, A07EA02 |
| Iloperidone | 1 | N05AX14 |
| Isosorbide | 1 | C01DA08, C01DA14, C01DA58, C05AE02 |
| Levocetirizine | 1 | R06AE09 |
| Loperamide | 1 | A07DA03, A07DA05, A07DA53 |
| Loratadine | 1 | R06AX13 |
| Metoprolol | 1 | C07AB02, C07BB02, C07BB52, C07CB02, C07FB02, C07FB13, C07FX03, C07FX05 |
| Morphine | 1 | N02AA01, N02AA04, N02AA51, N02AG01, R05DA01, R05DA05, A07DA52 |
| Nifedipine | 1 | C08CA05, C08CA55, C08GA01 |
| Paliperidone | 1 | N05AX13 |
| Prednisone | 1 | H02AB07, H02AB15, A07EA03 |
| Quinidine | 1 | C01BA01, C01BA13, C01BA51, C01BA71 |
| Ranitidine | 1 | A02BA02, A02BA07 |
| Risperidone | 1 | N05AX08 |
| Theophylline | 1 | R03DA04, R03DA54, R03DA74, R03DB04 |
| Trazodone | 1 | N06AX05 |
| Triamterene | 1 | C03DB02 |
| Venlafaxine | 1 | N06AX16 |
| Warfarin | 1 | B01AA03 |
| Amantadine | 2 | N04BB01 |
| Belladonna | 2 | A03BA04, A03CB02, A06AB30 |
| Carbamazepine | 2 | N03AF01 |
| Cyclobenzaprine | 2 | M03BX08 |
| Cyproheptadine | 2 | R06AX02 |
| Levomepromazine | 2 | N05AA02 |
| Loxapine | 2 | N05AH01 |
| Molindone | 2 | N05AE02 |
| Nefopam | 2 | N02BG06 |
| Oxcarbazepine | 2 | N03AF02 |
| Pethidine | 2 | N02AB02, N02AB52, N02AB72, N02AG03 |
| Pimozide | 2 | N05AG02 |
| Amitriptyline | 3 | N06AA09, N06CA01 |
| Amoxapine | 3 | N06AA17 |
| Atropine | 3 | A03BA01, A03CB03, A03BB02, A03BB06, A03CB04 |
| Benzatropine | 3 | N04AC01, N04AC30 |
| Brompheniramine | 3 | R06AB01, R06AB51, R06AB06, R06AB56 |
| Carbinoxamine | 3 | R06AA08 |
| Chlorphenamine | 3 | R06AB04, R06AB54, R06AB02, R06AB52 |
| Chlorpromazine | 3 | N05AA01 |
| Clemastine | 3 | R06AA04, R06AA54 |
| Clomipramine | 3 | N06AA04 |
| Clozapine | 3 | N05AH02 |
| Darifenacin | 3 | G04BD10 |
| Desipramine | 3 | N06AA01 |
| Dicycloverine | 3 | A03AA07 |
| Dimenhydrinate | 3 | R06AA11, R06AA61 |
| Diphenhydramine | 3 | R06AA02, R06AA52 |
| Doxepin | 3 | N06AA12 |
| Doxylamine | 3 | R06AA09, R06AA59 |
| Fesoterodine | 3 | G04BD11, G04BD13 |
| Flavoxate | 3 | G04BD02 |
| Hydroxyzine | 3 | N05BB01, N05BB51 |
| Hyoscyamine | 3 | A03BA03, A03CB31 |
| Imipramine | 3 | N06AA02, N06AA03 |
| Meclozine | 3 | R06AE05, R06AE55 |
| Methocarbamol | 3 | M03BA03, M03BA53, M03BA73 |
| Nortriptyline | 3 | N06AA10 |
| Olanzapine | 3 | N05AH03, N05AH53 |
| Orphenadrine | 3 | M03BC01, M03BC51, N04AB02 |
| Oxybutynin | 3 | G04BD04 |
| Paroxetine | 3 | N06AB05 |
| Perphenazine | 3 | N05AB03 |
| Promethazine | 3 | R06AD02, R06AD52, V03AB05, R06AD05, R06AD55 |
| Propantheline | 3 | A03AB05, A03CA34 |
| Propiverine | 3 | G04BD06 |
| Quetiapine | 3 | N05AH04 |
| Scopolamine | 3 | A04AD01, A04AD51, N05CM05, A03BB01, A03BB03, A03CB01, A03DB04 |
| Solifenacin | 3 | G04BD08, G04CA53 |
| Thioridazine | 3 | N05AC02 |
| Tolterodine | 3 | G04BD07 |
| Trifluoperazine | 3 | N05AB06 |
| Trihexyphenidyl | 3 | N04AA01 |
| Trimipramine | 3 | N06AA06 |
| Trospium | 3 | G04BD09, A03DA06 |

Abbreviation: ATC, Anatomical Therapeutic Chemical.

## Table S2. List of ICD-10 codes used to identify cardiovascular events

|  | **ICD-10 codes** |
| --- | --- |
| **Any cardiovascular event** | I00-I09, I11.0, I13.0, I13.2, I20-I89 |
| Myocardial infarction | I21, I22, I25.2 |
| Arrhythmias | I44.1, I44.2, I46, I47.0-I47.2, I48, I49.0, I49.5, I49.8 |
| Heart failure | I09.9, I11.0, I13.0, I13.2, I25.5, I42.0, I42.6-I42.9, I43, I50 |
| Cerebrovascular disease | I60-I69 |
| Arterial disease | I70-I74 |
| Venous thromboembolism | I26, I80-I82 |

Abbreviation: ICD-10, International Classification of Diseases 10th Revision.

## Table S3. List of ICD-10 codes and ATC codes used to define covariates

|  | **ICD-10 codes** | **ATC codes** |
| --- | --- | --- |
| **Surrogates for lifestyles** |  |  |
| Tobacco-related disorders | F17, T65.2, Z71.6, Z72.0 |  |
| Alcohol-related disorders | E24.4, F10, G31.2, G62.1, G72.1, I42.6, K29.2, K70, K86.0, O35.4, P04.3, Q86.0, T51.0, Y90, Y91, Z50.2, Z71.4, Z72.1 |  |
| Obesity | E65-E66 |  |
| **Physical conditions** |  |  |
| Hypertension | I10-I15 | C03, C07, C08, C09 |
| Diabetes mellitus | E10-E14 | A10A, A10B |
| Dyslipidemia | E78 |  |
| Any cancer (excl. non-melanoma skin cancer) | C00‐C97, excl. C44 |  |
| Rheumatic disease | M05, M06, M12.3, M07.0-M07.3, M08, M13, M30, M31.3-M31.6, M32-M34, M35.0, M35.1, M35.3, M45, M46 |  |
| Osteoarthritis | M15-M19 |  |
| Liver disease | B15-B19, K70-K77, R18, I85.0, I85.9, I98.2, I98.3 |  |
| Lung disease | J41-J47, J60-J70 |  |
| Kidney disease | N03.2-N03.7, N05.2-N05.7, N11, N18, N19, N25.0, I12.0, I13.1, Q61.1-Q61.4, Z49, Z94.0, Z99.2 |  |
| Urinary incontinence | N39.3, N39.4, R32 |  |
| Gastro-esophageal reflux disease/Esophagitis | K20, K21 |  |
| Peptic ulcer disease | K25-K28 |  |
| Inflammatory bowel disease | K50, K51, K52.3 |  |
| Irritable bowel syndrome | K58 |  |
| Vestibular disorders | H81 |  |
| Back pain | M54 |  |
| Chronic pain | F45.4, E10.4, E11.4, E12.4, E13.4, E14.4, B02.2, G50.0, G50.1, G53.0, M25.5, M50.1, M51.1, M54.1, M79.1, M79.2, M79.7, G63.1, G63.2, R52.1, R52.2 |  |
| **Neurological/psychiatric conditions** |  |  |
| Parkinson’s disease | G20 |  |
| Epilepsy | G40 |  |
| Migraine/Headache | G43, G44, R51 |  |
| Transient ischemic attack | G45 |  |
| Substance use disorder | F10-F19 |  |
| Psychotic disorders/Bipolar disorder | F20-F29, F30, F31 |  |
| Depression | F32, F33 |  |
| Anxiety disorders | F40, F41 |  |
| Stress-related disorders | F43 |  |
| Sleep disorders | F51, G47 |  |
| **Medications** |  |  |
| Renin-angiotensin system inhibitors |  | C09 |
| Beta-blockers |  | C07 |
| Calcium channel blockers |  | C08 |
| Diuretics |  | C03 |
| Statins |  | C10AA, C10B |
| Proton-pump inhibitors |  | A02BC |
| Antiplatelet drugs |  | B01AC |
| Nonsteroidal anti-inflammatory drugs |  | M01A |
| Opioids |  | N02A |
| Antiepileptics |  | N03A |
| Anti-Parkinson drugs |  | N04 |
| Antipsychotics |  | N05A |
| Anxiolytics, hypnotics, and sedatives |  | N05B, N05C |
| Antidepressants |  | N06A |
| Anti-addiction drugs |  | N07B |
| Abbreviations: ATC, Anatomical Therapeutic Chemical; ICD-10, International Classification of Diseases 10th Revision. | | |

Table S4. Association between baseline anticholinergic burden (annual DDDs) and incident cardiovascular events, using conventional multivariable Cox models

| Baseline exposure  **(**DDDs**)** | No. of events | Person-year | Incidence rate per 1000 person-years | Weighted HR (95% CI) | Conventional multivariable Cox models | | |
| --- | --- | --- | --- | --- | --- | --- | --- |
|  |  |  |  |  | Model 1 | Model 2 | Model 3 |
| 0 | 76,004 | 4,211,926 | 18.0 | 1.00 | 1.00 | 1.00 | 1.00 |
| 1-89 | 12,686 | 565,755 | 22.4 | 1.05 (1.02, 1.08) | 1.22 (1.19, 1.24) | 1.06 (1.04, 1.08) | 1.05 (1.03, 1.07) |
| 90-364 | 19,576 | 660,411 | 29.6 | 1.05 (1.01, 1.09) | 1.36 (1.34, 1.38) | 1.10 (1.08, 1.12) | 1.06 (1.04, 1.09) |
| ≥365 | 10,000 | 256,643 | 39.0 | 1.17 (1.08, 1.27) | 1.80 (1.76, 1.84) | 1.31 (1.28, 1.34) | 1.20 (1.16, 1.24) |

Abbreviations: CI, confidence interval; DDD, defined daily dose; HR, hazard ratio.

Model 1 was adjusted for age and sex.

Model 2 was additionally adjusted for socioeconomic status, healthcare utilization in the previous year, history of medical conditions, and use of medications other than anticholinergics at baseline.

Model 3 was additionally adjusted for past exposure to anticholinergic drugs.

## Table S5. Competing risk analysis of baseline anticholinergic burden (annual DDDs) and incident cardiovascular events

| Baseline exposure  (DDDs) | No. of events | Person-year | Incidence rate per 1000 person-years | Cause-specific HR (95% CI)^a,b^ | Subdistribution HR (95% CI)^a,c^ |
| --- | --- | --- | --- | --- | --- |
| **Incident CVD** |  |  |  |  |  |
| 0 | 76,004 | 4,211,926 | 18.0 | 1.00 | 1.00 |
| 1-89 | 12,686 | 565,755 | 22.4 | 1.05 (1.02, 1.08) | 1.03 (1.01, 1.06) |
| 90-364 | 19,576 | 660,411 | 29.6 | 1.05 (1.01, 1.09) | 1.03 (0.99, 1.07) |
| ≥365 | 10,000 | 256,643 | 39.0 | 1.17 (1.08, 1.27) | 1.12 (1.04, 1.21) |
| **Non-CVD mortality** |  |  |  |  |  |
| 0 | 45,045 | 4,557,795 | 9.9 | 1.00 | – |
| 1-89 | 8,561 | 623,301 | 13.7 | 1.04 (1.00, 1.08) | – |
| 90-364 | 12,765 | 744,608 | 17.1 | 1.05 (1.00, 1.10) | – |
| ≥365 | 7,215 | 296,900 | 24.3 | 1.29 (1.18, 1.41) | – |

Abbreviations: CI, confidence interval; CVD, cardiovascular disease; DDD, defined daily dose; HR, hazard ratio.

^a^ Model was weighted for baseline variables (age, sex, socioeconomic status, healthcare utilization in the previous year, history of medical conditions, use of medications other than anticholinergics), and pre-baseline exposure to anticholinergic drugs.

^b^ An inverse probability weighted Cox model was used.

^c^ An inverse probability weighted Fine-Gray model was used.

## Table S6. Characteristics according to the total ACB score at baseline

| Characteristics | Overall  N = 508,273 | 0  N = 365,398 | 1  N = 81,611 | 2–3  N = 39,179 | ≥4  N = 22,085 |
| --- | --- | --- | --- | --- | --- |
| **Age, years, median (IQR)** | 58.9 (51.6–66.6) | 58.0 (50.9–65.4) | 61.5 (53.9–69.8) | 61.5 (53.6–70.9) | 60.4 (52.7–69.6) |
| **Female** | 270,441 (53.2) | 182,777 (50.0) | 47,447 (58.1) | 25,118 (64.1) | 15,099 (68.4) |
| **Disposable income, ×100 SEK, median (IQR)^a^** | 2,080 (1,369–2,934) | 2,192 (1,452–3,049) | 1,933 (1,316–2,758) | 1,695 (1,235–2,497) | 1,509 (1,179–2,226) |
| **Educational attainment^a^** |  |  |  |  |  |
| Compulsory education | 104,848 (21.0) | 69,755 (19.4) | 19,439 (24.2) | 9,934 (25.9) | 5,720 (26.5) |
| Upper secondary education | 206,333 (41.3) | 147,140 (41.0) | 33,831 (42.1) | 16,067 (41.9) | 9,295 (43.0) |
| University/college | 187,964 (37.7) | 141,921 (39.6) | 27,105 (33.7) | 12,361 (32.2) | 6,577 (30.5) |
| **Marital status^a^** |  |  |  |  |  |
| Single | 199,270 (39.4) | 143,122 (39.4) | 29,593 (36.3) | 15,964 (40.8) | 10,591 (48.0) |
| Married | 268,191 (53.0) | 197,068 (54.2) | 43,412 (53.2) | 18,666 (47.7) | 9,045 (41.0) |
| Widowed | 38,717 (7.6) | 23,213 (6.4) | 8,540 (10.5) | 4,526 (11.6) | 2,438 (11.0) |
| **Any outpatient specialist visits in the past year** | 176,969 (34.8) | 103,646 (28.4) | 37,042 (45.4) | 21,531 (55.0) | 14,750 (66.8) |
| **Any hospitalization in the past year** | 35,643 (7.0) | 16,752 (4.6) | 8,543 (10.5) | 5,523 (14.1) | 4,825 (21.8) |
| **Tobacco-related disorders** | 4,961 (1.0) | 2,656 (0.7) | 1,105 (1.4) | 717 (1.8) | 483 (2.2) |
| **Alcohol-related disorders** | 12,564 (2.5) | 6,352 (1.7) | 2,167 (2.7) | 1,968 (5.0) | 2,077 (9.4) |
| **Obesity diagnosis** | 12,933 (2.5) | 6,229 (1.7) | 3,430 (4.2) | 1,940 (5.0) | 1,334 (6.0) |
| **Physical conditions** |  |  |  |  |  |
| Hypertension | 138,366 (27.2) | 59,896 (16.4) | 48,056 (58.9) | 19,237 (49.1) | 11,177 (50.6) |
| Chronic pain | 84,731 (16.7) | 52,211 (14.3) | 16,746 (20.5) | 9,448 (24.1) | 6,326 (28.6) |
| Back pain | 66,932 (13.2) | 40,274 (11.0) | 13,524 (16.6) | 7,746 (19.8) | 5,388 (24.4) |
| Osteoarthritis | 49,327 (9.7) | 29,481 (8.1) | 10,875 (13.3) | 5,523 (14.1) | 3,448 (15.6) |
| Cancer | 34,681 (6.8) | 20,902 (5.7) | 7,331 (9.0) | 4,004 (10.2) | 2,444 (11.1) |
| Diabetes mellitus | 33,878 (6.7) | 17,732 (4.9) | 8,904 (10.9) | 4,593 (11.7) | 2,649 (12.0) |
| Lung disease | 30,655 (6.0) | 16,651 (4.6) | 7,173 (8.8) | 4,064 (10.4) | 2,767 (12.5) |
| Dyslipidemia | 29,516 (5.8) | 16,060 (4.4) | 8,136 (10.0) | 3,491 (8.9) | 1,829 (8.3) |
| Kidney disease | 24,972 (4.9) | 10,954 (3.0) | 7,228 (8.9) | 4,347 (11.1) | 2,443 (11.1) |
| Rheumatic disease | 17,907 (3.5) | 10,629 (2.9) | 3,823 (4.7) | 2,132 (5.4) | 1,323 (6.0) |
| Gastroesophageal reflux disease | 14,326 (2.8) | 8,009 (2.2) | 3,138 (3.8) | 1,802 (4.6) | 1,377 (6.2) |
| Urinary incontinence | 11,983 (2.4) | 5,928 (1.6) | 2,067 (2.5) | 2,094 (5.3) | 1,894 (8.6) |
| Vestibular disorder | 11,329 (2.2) | 7,072 (1.9) | 2,191 (2.7) | 1,295 (3.3) | 771 (3.5) |
| Irritable bowel syndrome | 8,559 (1.7) | 4,621 (1.3) | 1,694 (2.1) | 1,257 (3.2) | 987 (4.5) |
| Liver disease | 7,431 (1.5) | 4,272 (1.2) | 1,379 (1.7) | 971 (2.5) | 809 (3.7) |
| Peptic ulcer disease | 5,089 (1.0) | 2,821 (0.8) | 1,095 (1.3) | 653 (1.7) | 520 (2.4) |
| Inflammatory bowel disease | 4,810 (0.9) | 2,940 (0.8) | 1,023 (1.3) | 491 (1.3) | 356 (1.6) |
| **Neurological/psychiatric conditions** |  |  |  |  |  |
| Depression | 29,792 (5.9) | 13,871 (3.8) | 5,803 (7.1) | 5,222 (13.3) | 4,896 (22.2) |
| Migraine/headache | 29,395 (5.8) | 16,946 (4.6) | 6,003 (7.4) | 3,641 (9.3) | 2,805 (12.7) |
| Sleep disorders | 22,578 (4.4) | 12,005 (3.3) | 4,655 (5.7) | 3,412 (8.7) | 2,506 (11.3) |
| Stress-related disorders | 20,637 (4.1) | 11,891 (3.3) | 3,795 (4.7) | 2,794 (7.1) | 2,157 (9.8) |
| Substance use disorder | 17,780 (3.5) | 8,950 (2.4) | 3,235 (4.0) | 2,763 (7.1) | 2,832 (12.8) |
| Anxiety disorders | 16,668 (3.3) | 6,527 (1.8) | 3,230 (4.0) | 3,351 (8.6) | 3,560 (16.1) |
| Psychotic disorders | 6,973 (1.4) | 1,840 (0.5) | 1,023 (1.3) | 1,691 (4.3) | 2,419 (11.0) |
| Transient ischemic attack | 4,213 (0.8) | 2,254 (0.6) | 1,107 (1.4) | 528 (1.3) | 324 (1.5) |
| Epilepsy | 3,908 (0.8) | 1,510 (0.4) | 490 (0.6) | 1,299 (3.3) | 609 (2.8) |
| Parkinson’s disease | 1,431 (0.3) | 726 (0.2) | 233 (0.3) | 273 (0.7) | 199 (0.9) |
| **Medications^b^** |  |  |  |  |  |
| Number of distinct drugs, median (IQR) | 2 (1–5) | 2 (0–4) | 4 (2–7) | 6 (3–10) | 8 (5–13) |
| Nonsteroidal anti-inflammatory drugs | 100,992 (19.9) | 58,430 (16.0) | 22,477 (27.5) | 11,991 (30.6) | 8,094 (36.6) |
| Anxiolytics, hypnotics, and sedatives | 72,655 (14.3) | 31,194 (8.5) | 16,721 (20.5) | 13,187 (33.7) | 11,553 (52.3) |
| Renin-angiotensin system inhibitors | 68,793 (13.5) | 34,925 (9.6) | 20,408 (25.0) | 8,968 (22.9) | 4,492 (20.3) |
| Statins | 54,349 (10.7) | 27,414 (7.5) | 15,903 (19.5) | 7,233 (18.5) | 3,799 (17.2) |
| Proton-pump inhibitors | 45,767 (9.0) | 21,105 (5.8) | 11,230 (13.8) | 7,510 (19.2) | 5,922 (26.8) |
| Antidepressants | 37,880 (7.5) | 16,435 (4.5) | 8,066 (9.9) | 6,981 (17.8) | 6,398 (29.0) |
| Diuretics | 37,165 (7.3) | 16,626 (4.6) | 12,786 (15.7) | 4,910 (12.5) | 2,843 (12.9) |
| Calcium channel blockers | 35,143 (6.9) | 13,799 (3.8) | 13,350 (16.4) | 5,321 (13.6) | 2,673 (12.1) |
| Opioids | 30,946 (6.1) | 14,007 (3.8) | 7,221 (8.8) | 5,280 (13.5) | 4,438 (20.1) |
| Antiplatelet drugs | 30,053 (5.9) | 12,889 (3.5) | 9,853 (12.1) | 4,813 (12.3) | 2,498 (11.3) |
| Beta-blockers | 11,728 (2.3) | 6,361 (1.7) | 2,498 (3.1) | 1,596 (4.1) | 1,273 (5.8) |
| Antiepileptics | 7,692 (1.5) | 2,295 (0.6) | 1,334 (1.6) | 1,831 (4.7) | 2,232 (10.1) |
| Anti-addiction drugs | 7,413 (1.5) | 3,528 (1.0) | 1,475 (1.8) | 1,172 (3.0) | 1,238 (5.6) |
| Anti-Parkinson drugs | 4,782 (0.9) | 1,865 (0.5) | 928 (1.1) | 915 (2.3) | 1,074 (4.9) |
| Antipsychotics | 4,650 (0.9) | 1,351 (0.4) | 686 (0.8) | 1,068 (2.7) | 1,545 (7.0) |

Abbreviations: ACB, Anticholinergic Cognitive Burden; IQR, interquartile range.

Frequency with percentage was reported unless otherwise specified.

^a^ Disposable income and marital status were missing for 2,095 (0.4%) individuals; educational attainment was missing for 9,128 (1.8%) individuals.

^b^ Drugs listed in the Anticholinergic Cognitive Burden scale were excluded from the covariate definition.

## Table S7. Association between anticholinergic burden (ACB score) and incident cardiovascular events

|  | No. of events | Person-year | Incidence rate per 1000 person-years | Weighted HR (95% CI)^a^ |
| --- | --- | --- | --- | --- |
| **Baseline exposure (score)** |  |  |  |  |
| 0 | 76,004 | 4,211,926 | 18.0 | 1.00 |
| 1 | 23,534 | 865,353 | 27.2 | 1.03 (1.01, 1.06) |
| 2-3 | 11,961 | 397,930 | 30.1 | 1.03 (0.99, 1.07) |
| ≥4 | 6,767 | 219,526 | 30.8 | 1.18 (1.11, 1.25) |
| **Time-varying exposure (score)** |  |  |  |  |
| 0 | 62,942 | 3,927,587 | 16.0 | 1.00 |
| 1 | 28,159 | 1,000,228 | 28.2 | 1.29 (1.22, 1.36) |
| 2-3 | 17,860 | 492,207 | 36.3 | 1.33 (1.25, 1.42) |
| ≥4 | 9,305 | 274,713 | 33.9 | 1.37 (1.25, 1.50) |

Abbreviations: ACB, Anticholinergic Cognitive Burden; CI, confidence interval; HR, hazard ratio.

^a^ Model was weighted for age, sex, socioeconomic status, healthcare utilization, medical conditions, use of medications other than anticholinergics, and pre-baseline exposure to anticholinergic drugs.

## Table S8. Association between time-varying anticholinergic burden (annual DDDs) and incident cardiovascular events, stratified by sex

| Annual DDDs | No. of events | Person-year | Incidence rate per 1000 person-years | Weighted HR (95% CI)^a^ |
| --- | --- | --- | --- | --- |
| **Women** |  |  |  |  |
| 0 | 25,645 | 2,003,383 | 12.8 | 1.00 |
| 1-89 | 7,821 | 381,724 | 20.5 | 1.18 (1.13, 1.23) |
| 90-364 | 13,545 | 497,400 | 27.2 | 1.34 (1.30, 1.38) |
| ≥365 | 8,254 | 205,516 | 40.2 | 1.78 (1.72, 1.85) |
| **Men** |  |  |  |  |
| 0 | 37,297 | 1,924,204 | 19.4 | 1.00 |
| 1-89 | 7,345 | 240,612 | 30.5 | 1.15 (1.10, 1.20) |
| 90-364 | 11,890 | 313,592 | 37.9 | 1.28 (1.24, 1.32) |
| ≥365 | 6,469 | 128,305 | 50.4 | 1.64 (1.58, 1.70) |

Abbreviations: CI, confidence interval; DDD, defined daily dose; HR, hazard ratio.

P for interaction <0.001.

^a^ Model was weighted for age, sex, socioeconomic status, healthcare utilization, medical conditions, use of medications other than anticholinergics, and pre-baseline exposure to anticholinergic drugs.

## Table S9. Association between time-varying anticholinergic burden (annual DDDs) and incident cardiovascular events, stratified by age groups at baseline

| Annual DDDs | No. of events | Person-year | Incidence rate per 1000 person-years | Weighted HR (95% CI)^a^ |
| --- | --- | --- | --- | --- |
| **<55 years** |  |  |  |  |
| 0 | 12,791 | 1,738,806 | 7.4 | 1.00 |
| 1-89 | 2,622 | 250,561 | 10.5 | 1.16 (1.08, 1.24) |
| 90-364 | 3,219 | 260,111 | 12.4 | 1.28 (1.21, 1.36) |
| ≥365 | 1,935 | 108,696 | 17.8 | 1.72 (1.61, 1.85) |
| **55–64 years** |  |  |  |  |
| 0 | 20,126 | 1,381,425 | 14.6 | 1.00 |
| 1-89 | 4,422 | 212,997 | 20.8 | 1.17 (1.11, 1.23) |
| 90-364 | 6,915 | 294,376 | 23.5 | 1.31 (1.26, 1.37) |
| ≥365 | 3,945 | 117,508 | 33.6 | 1.73 (1.65, 1.82) |
| **≥65 years** |  |  |  |  |
| 0 | 30,025 | 807,356 | 37.2 | 1.00 |
| 1-89 | 8,122 | 158,779 | 51.2 | 1.16 (1.11, 1.21) |
| 90-364 | 15,301 | 256,505 | 59.7 | 1.31 (1.27, 1.35) |
| ≥365 | 8,843 | 107,616 | 82.2 | 1.70 (1.65, 1.76) |

Abbreviations: CI, confidence interval; DDD, defined daily dose; HR, hazard ratio.

P for interaction <0.001.

^a^ Model was weighted for age, sex, socioeconomic status, healthcare utilization, medical conditions, use of medications other than anticholinergics, and pre-baseline exposure to anticholinergic drugs.


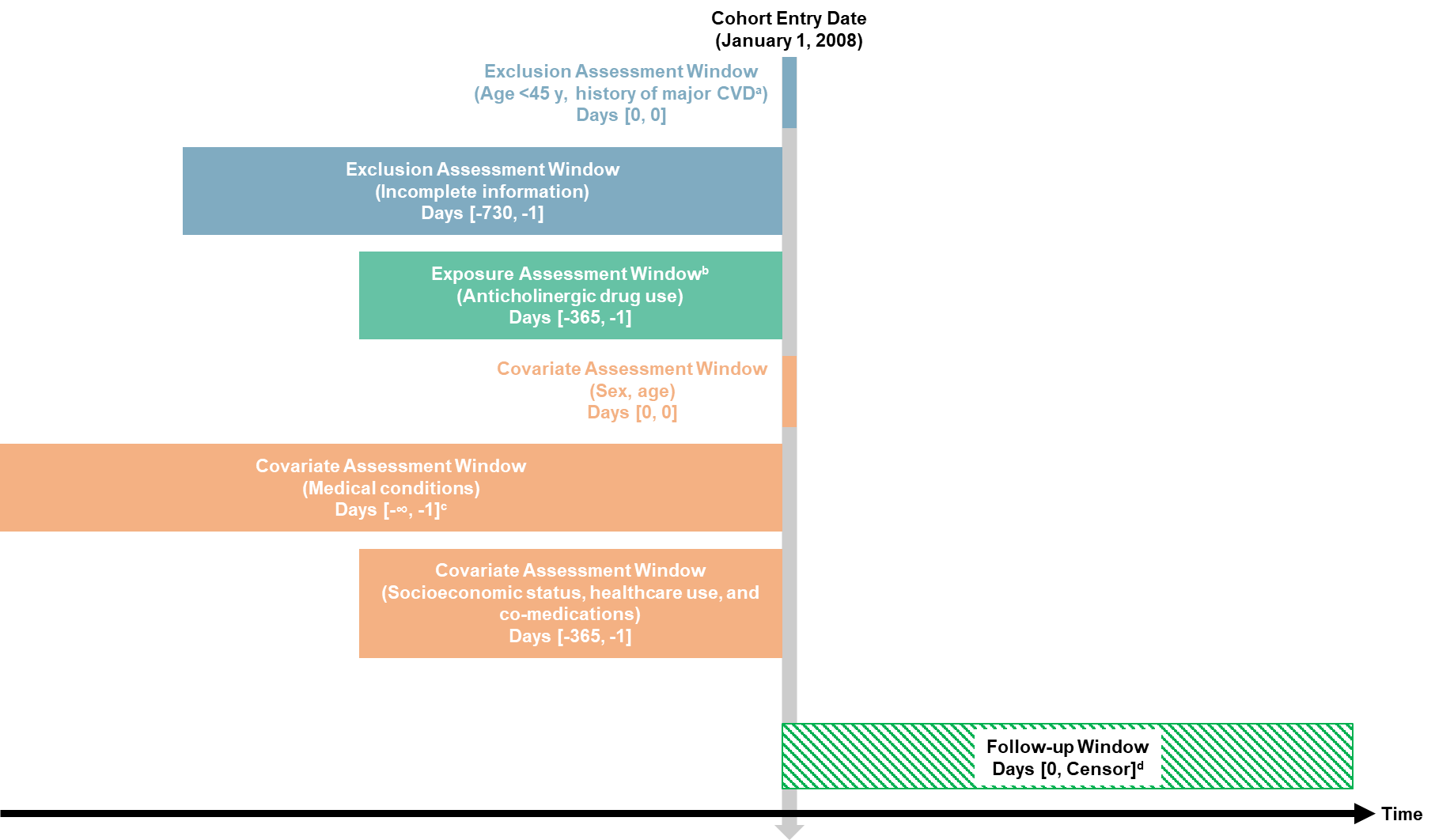


## Figure S1. Graphical depiction of the study design

^a^ Individuals with hypertension but without hypertensive heart failure or any other cardiovascular diseases were eligible for inclusion.

^b^ Anticholinergic burden was updated using one-year sliding windows when analyzing time-varying exposure.

^c^ Healthcare data from 1997 onward were available to ascertain history of medical conditions.

^d^ Follow-up was censored at the earliest of outcome, death, emigration out of the region, or end of study period.


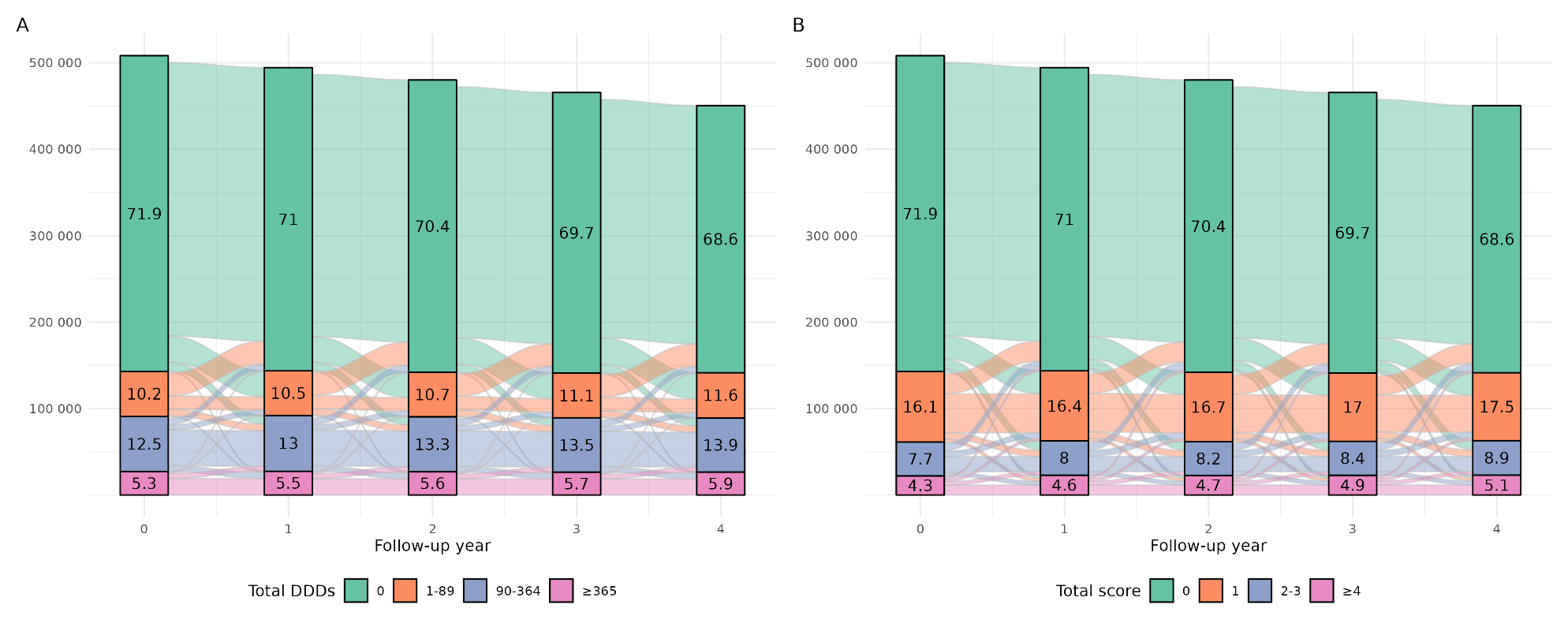


## Figure S2. Alluvial plots illustrating the changes in drug exposure categories over time

Abbreviation: DDD, defined daily dose.

The overall height of each bar represents the number of individuals under follow-up in each year. Values within the bars indicate the proportion of exposure categories among individuals under follow-up.


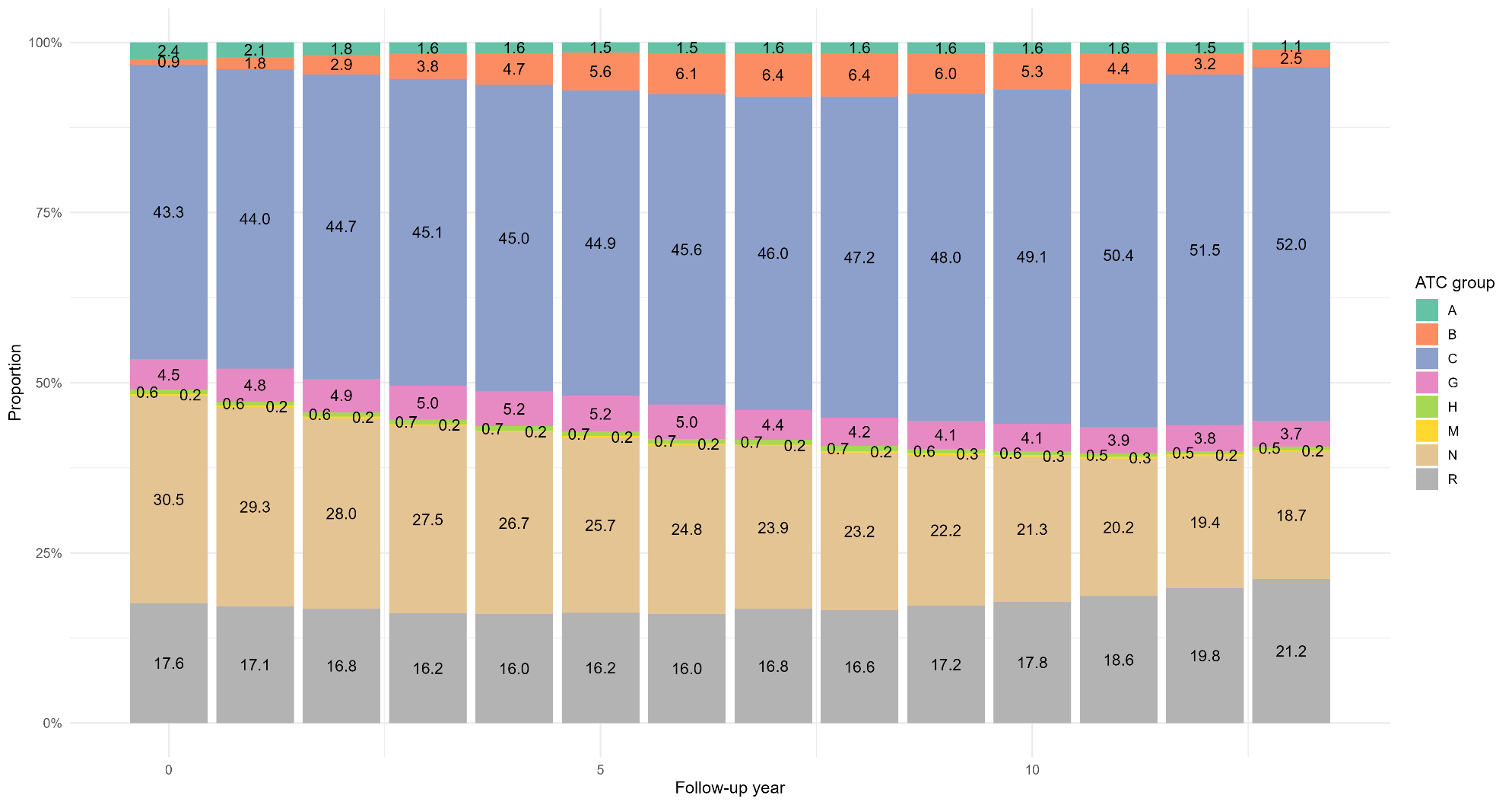


## Figure S3. Distribution of population total DDDs of anticholinergic drugs by ATC groups

Abbreviations: ATC, Anatomical Therapeutic Chemical; DDD, defined daily dose.

ATC groups: A, Alimentary tract and metabolism; B, Blood and blood forming organs; C, Cardiovascular system; G, Genito urinary system and sex hormones; H, Systemic hormonal preparations, excl. sex hormones and insulins; M, Musculo-skeletal system; N, Nervous system; R, Respiratory system.


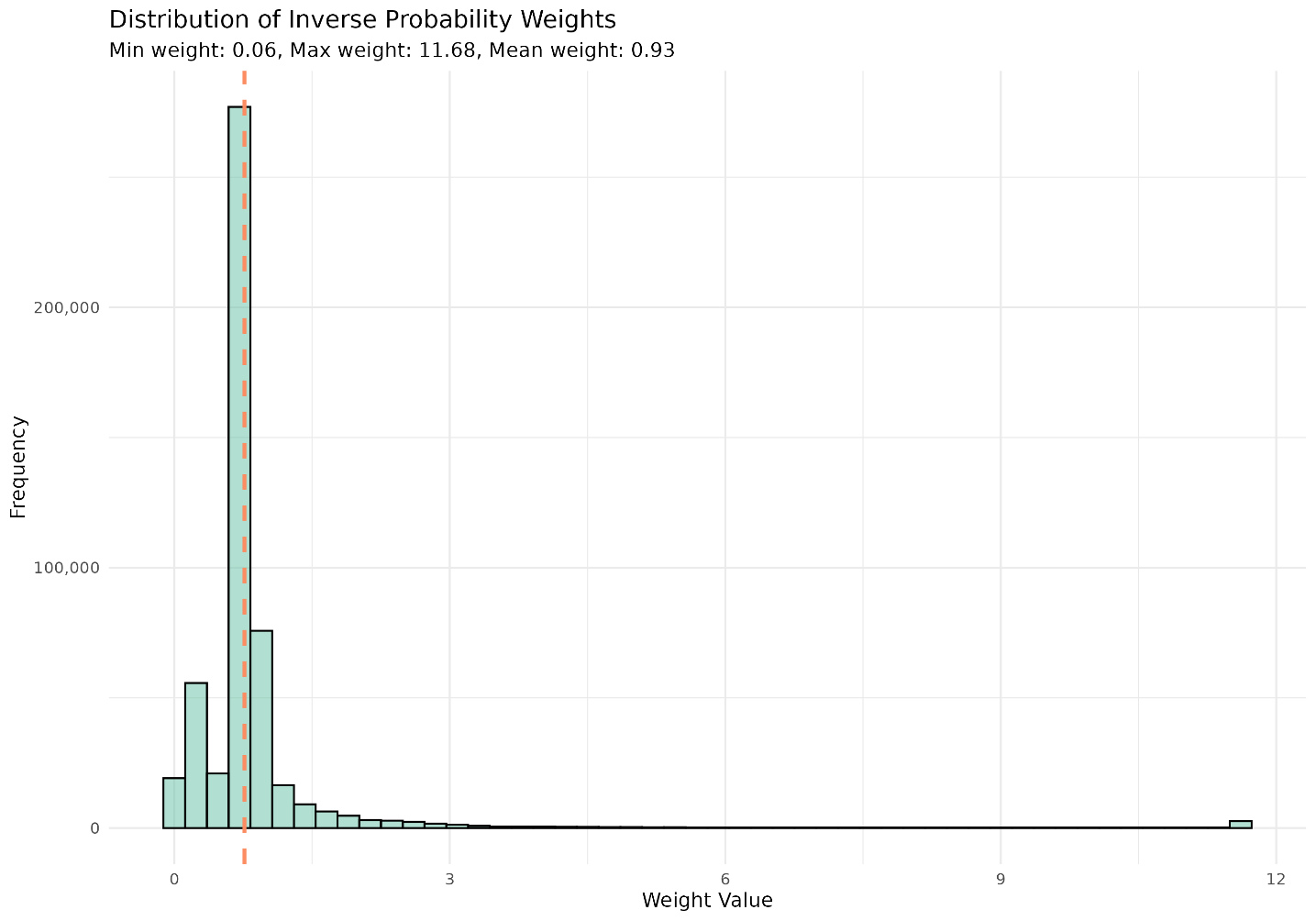


## Figure S4. Distribution of baseline inverse probability weights

Stabilized inverse probability weights are truncated at the 0.5th and 99.5th percentiles. The dotted line represents the mean value.

**
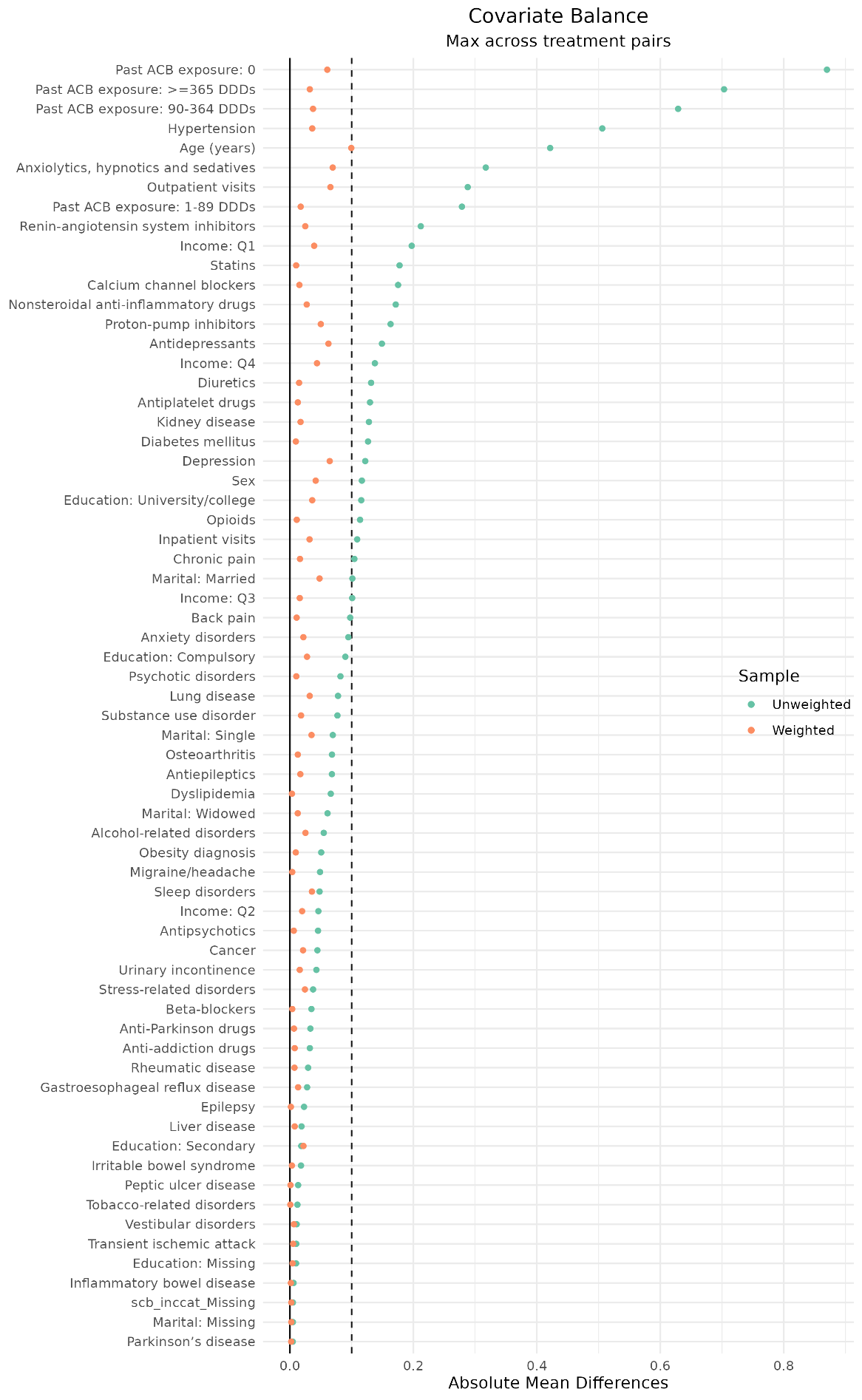
**

## Figure S5. Covariate balance before and after baseline inverse probability weighting


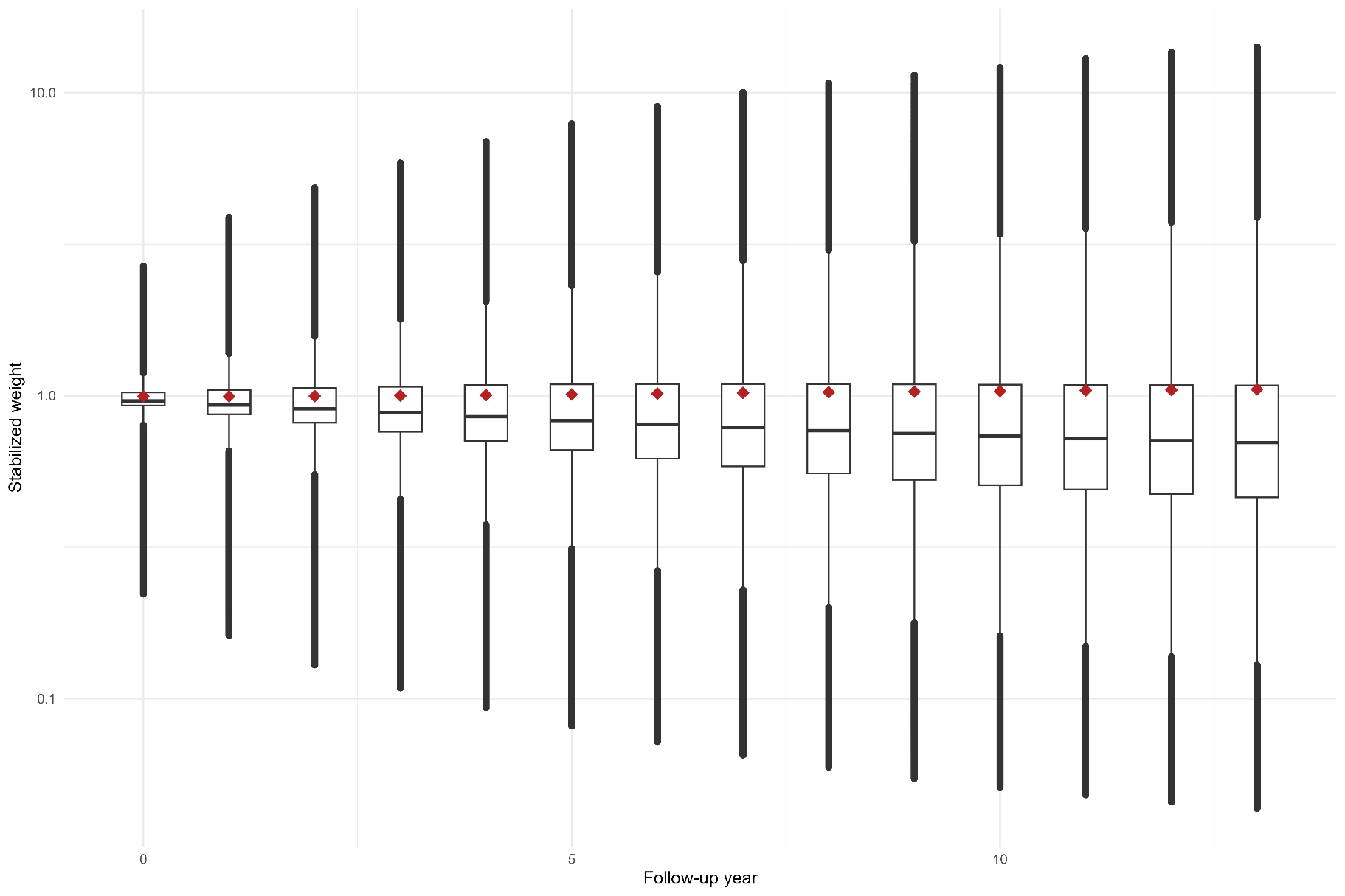


## Figure S6. Distribution of time-varying inverse probability weights

Dark red points indicate the mean stabilized inverse probability weights by each year.

| \|  \| No. of events \| Person-year \| Incidence rate per 1000 person-years \| Weighted HR (95% CI)^a^ \| \| --- \| --- \| --- \| --- \| --- \| \| **Myocardial infarction** \|  \|  \|  \|  \| \| 0 \| 12,412 \| 4,070,706 \| 3.0 \| 1.00 \| \| 1 \| 5,338 \| 1,126,501 \| 4.7 \| 1.12 (1.06, 1.18) \| \| 2-3 \| 3,563 \| 599,855 \| 5.9 \| 1.21 (1.14, 1.29) \| \| ≥4 \| 1,848 \| 321,882 \| 5.7 \| 1.37 (1.24, 1.50) \| \| **Cerebrovascular disease** \|  \|  \|  \|  \| \| 0 \| 16,002 \| 4,040,372 \| 4.0 \| 1.00 \| \| 1 \| 7,236 \| 1,134,789 \| 6.4 \| 1.06 (1.01, 1.11) \| \| 2-3 \| 5,158 \| 598,342 \| 8.6 \| 1.24 (1.17, 1.31) \| \| ≥4 \| 2,665 \| 318,469 \| 8.4 \| 1.32 (1.22, 1.43) \| \| **Arrhythmias** \|  \|  \|  \|  \| \| 0 \| 17,868 \| 4,051,341 \| 4.4 \| 1.00 \| \| 1 \| 10,713 \| 1,119,672 \| 9.6 \| 1.44 (1.38, 1.50) \| \| 2-3 \| 8,065 \| 568,175 \| 14.2 \| 1.86 (1.78, 1.94) \| \| ≥4 \| 3,496 \| 309,983 \| 11.3 \| 1.70 (1.59, 1.82) \| \| **Heart failure** \|  \|  \|  \|  \| \| 0 \| 9,810 \| 4,080,153 \| 2.4 \| 1.00 \| \| 1 \| 6,869 \| 1,149,721 \| 6.0 \| 1.51 (1.43, 1.59) \| \| 2-3 \| 6,667 \| 594,936 \| 11.2 \| 2.07 (1.96, 2.19) \| \| ≥4 \| 3,223 \| 319,048 \| 10.1 \| 2.05 (1.90, 2.22) \| \| **Artery disease** \|  \|  \|  \|  \| \| 0 \| 4,623 \| 4,078,612 \| 1.1 \| 1.00 \| \| 1 \| 2,622 \| 1,160,264 \| 2.3 \| 1.10 (1.01, 1.19) \| \| 2-3 \| 1,907 \| 618,575 \| 3.1 \| 1.25 (1.13, 1.37) \| \| ≥4 \| 995 \| 328,826 \| 3.0 \| 1.43 (1.25, 1.64) \| \| **Venous thromboembolism** \|  \|  \|  \|  \| \| 0 \| 6,879 \| 4,073,201 \| 1.7 \| 1.00 \| \| 1 \| 3,215 \| 1,157,107 \| 2.8 \| 1.22 (1.13, 1.31) \| \| 2-3 \| 2,083 \| 616,243 \| 3.4 \| 1.21 (1.10, 1.32) \| \| ≥4 \| 1,250 \| 326,958 \| 3.8 \| 1.22 (1.07, 1.38) \| | 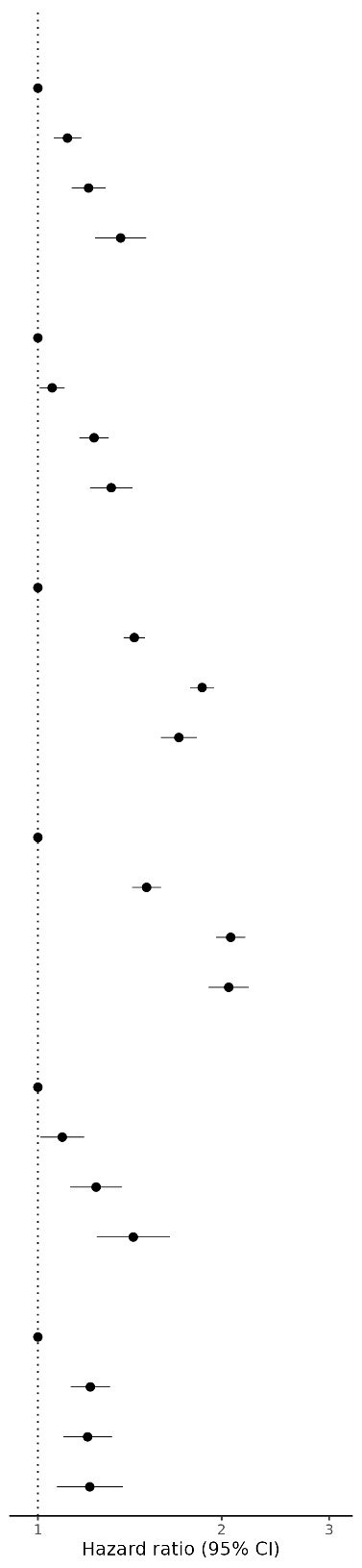 |
| --- | --- | --- | --- | --- | --- | --- | --- | --- | --- | --- | --- | --- | --- | --- | --- | --- | --- | --- | --- | --- | --- | --- | --- | --- | --- | --- | --- | --- | --- | --- | --- | --- | --- | --- | --- | --- | --- | --- | --- | --- | --- | --- | --- | --- | --- | --- | --- | --- | --- | --- | --- | --- | --- | --- | --- | --- | --- | --- | --- | --- | --- | --- | --- | --- | --- | --- | --- | --- | --- | --- | --- | --- | --- | --- | --- | --- | --- | --- | --- | --- | --- | --- | --- | --- | --- | --- | --- | --- | --- | --- | --- | --- | --- | --- | --- | --- | --- | --- | --- | --- | --- | --- | --- | --- | --- | --- | --- | --- | --- | --- | --- | --- | --- | --- | --- | --- | --- | --- | --- | --- | --- | --- | --- | --- | --- | --- | --- | --- | --- | --- | --- | --- | --- | --- | --- | --- | --- | --- | --- | --- | --- | --- | --- | --- | --- | --- | --- | --- | --- | --- | --- | --- | --- | --- | --- | --- |

## Figure S7. Associations between time-varying anticholinergic burden (ACB score) and specific cardiovascular events

Abbreviations: ACB, Anticholinergic Cognitive Burden; CI, confidence interval; HR, hazard ratio.

^a^ Models were weighted for age, sex, socioeconomic status, healthcare utilization, medical conditions, use of medications other than anticholinergics, and pre-baseline exposure to anticholinergic drugs.
